# The complex journey of targeting RAS in oncology

**DOI:** 10.1186/s12885-025-14033-y

**Published:** 2025-07-01

**Authors:** Katarzyna Wasiak, Damian Ciunowicz, Amelia Kierasińska-Kałka, Marta Węgierska, Marcin Pacholczyk, Piotr Rieske, Ewelina Stoczyńska-Fidelus

**Affiliations:** 1 Department of Research and Development, Personather Ltd., Inwestycyjna 7, Konstantynow Lodzki, 95 - 050 Poland; 2 Department of Tumor Biology, Chair of Medical Biology, Medical University of Lodz, Zeligowskiego 7/9 St., Lodz, 90 - 752 Poland; 3 Department of Systems Biology and Engineering, Silesian University of Technology, Akademicka 16, Gliwice, 44 - 100 Poland; 4 Department of Molecular Biology, Chair of Medical Biology, Medical University of Lodz, Zeligowskiego 7/9 St., Lodz, 90 - 752 Poland

**Keywords:** RAS proteins, KRAS, HRAS, NRAS, Oncogene, Cancer, Therapy, Protein structure, Drug discovery, Senescence

## Abstract

Given the prevalence of RAS mutations in various cancers, personalized therapeutic approaches, guided by molecular markers, are essential. Farnesyltransferase inhibitors (FTIs) have emerged as potential therapeutic options; however, they also face obstacles such as toxicity and limited efficacy. Alternative strategies, such as direct inhibitors combined with pathway modulators, RNA interference, and gene-editing technologies, are under clinical investigation. The targeting of RAS, complicated by its structural nuances, particularly in the G domain, has advanced with the identification of druggable pockets such as the SW-II pocket. This breakthrough has led to the development of targeted therapeutics, such as sotorasib and adagrasib, for KRAS G12C-mutated non-small cell lung cancer (NSCLC). However, these advancements face challenges, including adaptive resistance and the necessity for isoform selectivity. New inhibitors, such as LY3537982 or GDC-6036, are promising, but achieving effective and selective RAS inhibition remains a significant challenge. Additionally, clinical trials have highlighted variability in patient responses, attributing limited treatment efficacy to resistance mechanisms, including on-target mutations and off-target pathway activations. Finally, the RAS oncogene, traditionally viewed as predominantly pro-cancerous, plays a complex role in oncogenesis, with recent evidence suggesting context-dependent effects, such as inducing senescence in certain cells. This shift in understanding underscores the therapeutic potential of manipulating the interplay between RAS and TP53 mutations in cancer. In conclusion, the complexity of effectively targeting the RAS-RAF-ERK pathway is exacerbated by the diverse resistance mechanisms. Challenges such as off-target effects and delivery issues remain significant barriers in the introduction of effective therapies based on RAS inhibitors. This overview highlights the evolving nature of targeting RAS in cancer therapy.

## Introduction

**Fig. 1:**
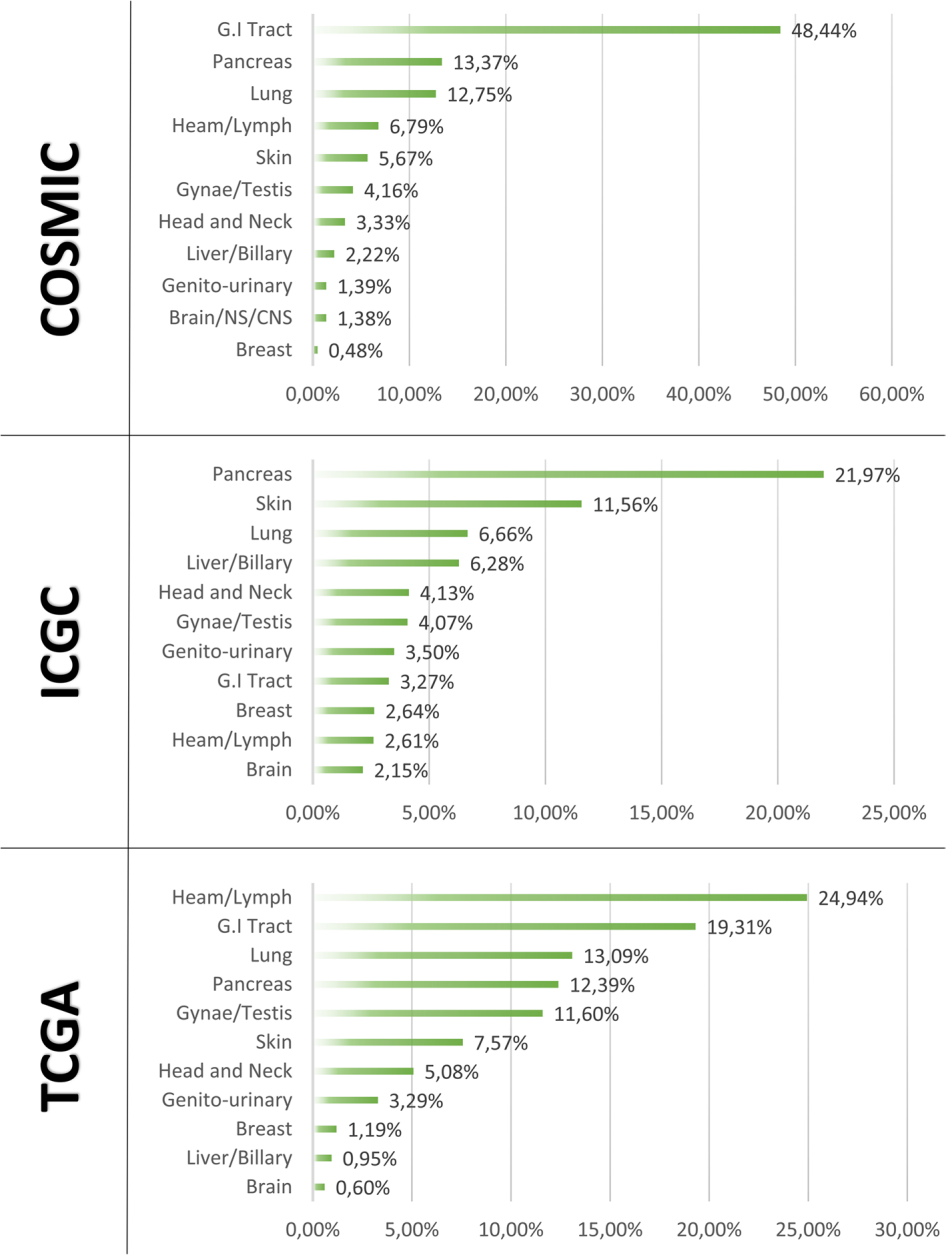
Distribution of mutation percentages for each RAS mutation subtype according to the analyzed database [1, 6, 7]

The RAS family of small GTPases has long been the subject of intense research in oncology due to its central role in the development and progression of various cancer types. Oncogenic mutations of RAS, particularly KRAS, are found in a significant proportion of human cancers, including gastrointestinal (< 50%), pancreatic (13.37%), and lung (12.75%) cancers (Fig. 1) [1]. Across all databases, KRAS was the predominant variant affecting patients, accounting for 63–82% of the cohort. Conversely, HRAS constituted the smallest proportion, representing 4–13% of the cohort, depending on the database. Among the three members of the RAS gene family, the most common mutations are G12, G13, and Q61. However, the G12 mutation was the most common among KRAS and Q61 mutations in NRAS. For HRAS, the results varied among the databases.

These mutations impair RAS intrinsic GTPase activity and its interaction with GTPase-activating proteins (GAPs), shifting the RAS equilibrium in favor of the GTP-bound (ON) state. This results in constitutive activation of downstream pathways, which in turn drive tumorigenesis by uncontrolled cell proliferation, evasion of apoptosis, and the promotion of a pro-tumorigenic microenvironment [2–4].

Despite extensive research over the past three decades, effective therapeutic strategies against oncogenic RAS remained elusive for a long time. Several structural and biochemical characteristics of the RAS protein have posed challenges in the development of small-molecule inhibitors, including the lack of a deep hydrophobic drug-binding pocket or groove, its ultrahigh (picomolar) affinity for guanine nucleotide substrates, and the high intracellular concentrations of GTP. Additionally, oncogenic and wild-type RAS proteins differ minimally in structure, with the former possessing only a one amino acid missense mutation in the G-domain [5]. Given these challenges, researchers have begun exploring alternative approaches to targeting RAS-driven cancers. To date, several innovative strategies have been proposed, including novel direct inhibitors for G12C mutants, an expanded portfolio of G12D-specific inhibitors, RAS degraders, adoptive cell therapy, immunotherapy, RNAi-based technologies, and gene-editing therapies.

Since RAS is no longer regarded as a negative biomarker or an “undruggable” oncoprotein, we aim to explore the advancements that have shaped its current perception as a demanding yet achievable target for effective therapy. For a long time, efforts were made to target RAS in its GTP-bound form, competing with high GTP levels in the cytoplasm. This paradigm shifted once Kevin Shokat and his team identified covalent KRAS G12C inhibitors that bind to KRAS in its off-state. Currently, four KRAS G12C inhibitors have been approved, and numerous RAS and KRAS inhibitors have advanced to clinical trials, marking significant progress in clinical research. This raises crucial questions: How have the latest RAS-targeted small molecules performed in patients? To what extent has drug resistance posed a challenge for the scientific community? And, given recent findings on the effectiveness of RAS inhibitors, is there a real chance of developing a long-term, effective targeted therapy for RAS-mutant cancers?

At the same time, emerging insights into RAS biology deserve attention. Beyond its role as an oncoprotein, RAS functions are intricately shaped by the cell's genetic context, as seen in RAS-induced senescence. This raises a crucial question: Can these findings be translated into clinical applications? In this review, we explore potential answers to this and related questions.

### RAS oncogene and tumorigenesis. From senescence to intensification of proliferation

**Fig. 2:**
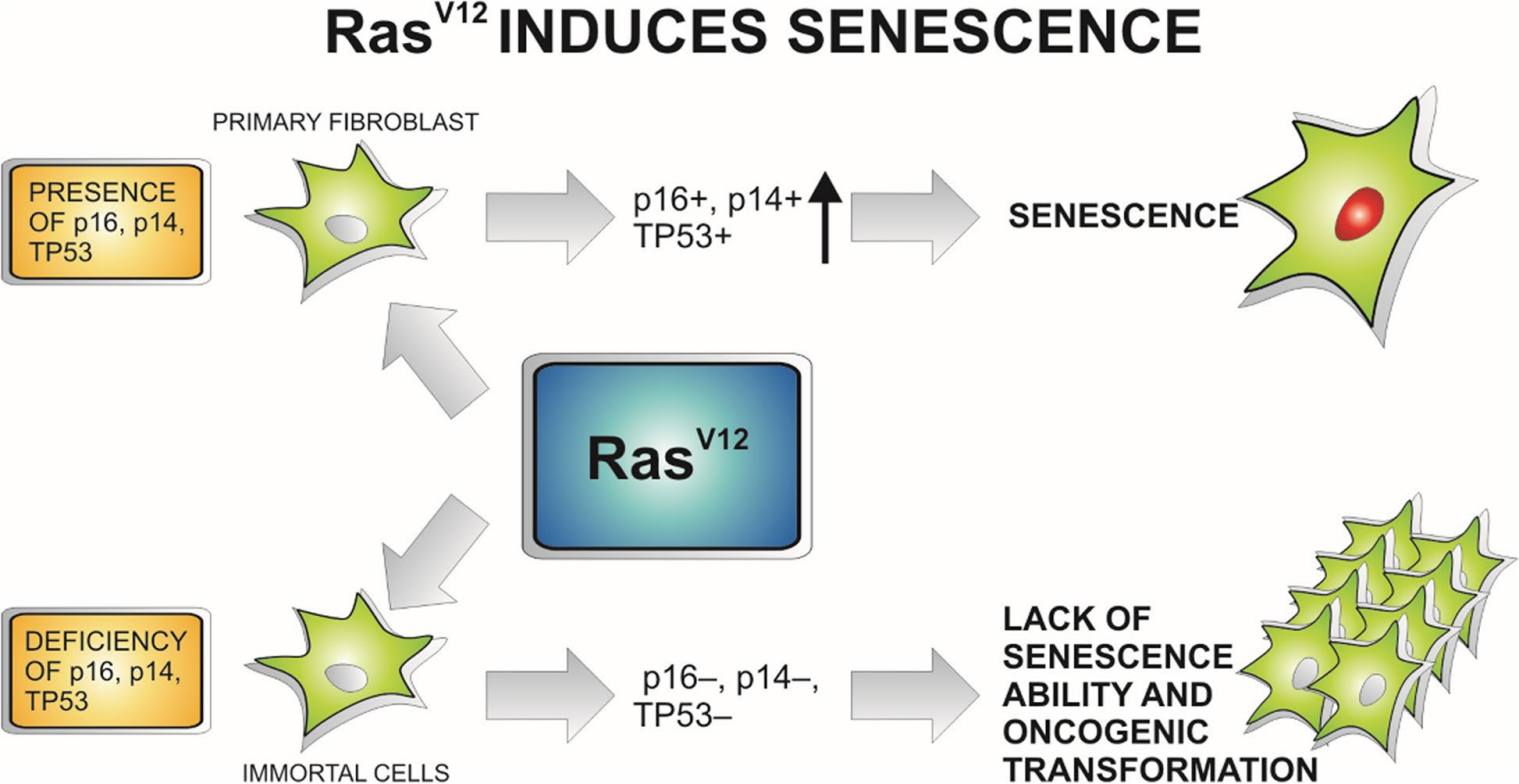
Ras G12V-induced senescence

It is commonly believed that the RAS oncogene always has procancer activity [8]. RAS activates a network of signaling pathways. Most important are RAF-MEK (PI3K)/AKT and protein kinase C (PKC). Other RAS-regulated pathways were also identified, including RalGDs/Ral, TIAM1/Rac, p190/Rho, PLC-ε, and RIN. Normally, thanks to the GEF protein, RAS binds to GTP and transduces signals to BRAF, PI3K, etc. However, other proteins such as GTPase-activating proteins (GAPs) induce the hydrolysis of GTP to GDP, thereby deactivating RAS. When RAS is mutated, hydrolysis of GTP to GDP is dramatically delayed and the signal is transduced for a much longer time [9–11]. Due to the virtually complete loss of GTPase activity, the signal should be chronically transduced, mainly to BRAF and AKT proteins, and this process is thought to translate into the increased proliferation of cancer cells [12]. Ras/Raf/ERK pathway-induced senescence correlates with the induction of genes such as p16/INK4A, p53, p21, and p14-p19/ARF [13]. In fact, the situation is significantly more complicated. The consequences of RAS oncogene mutations depend on the molecular context in which RAS mutants act. Surprisingly, RAS mutants, such as RAS G12V, in mouse embryonic fibroblasts (MEFs) induce senescence rather than proliferation [14]. Based on studies on mouse fibroblasts genetically inactivated for TP53, it has been indicated that TP53 mutations alter the effect of the RAS G12V protein on proliferation [15]. In human esophageal keratinocytes TP53 mutation does not stop the prosenescent action of RAS G12V [16]. In contrast, normal human foreskin fibroblasts (HFFs) are resistant to this type of action of RAS mutants even during normal TP53 operations [17]. Moreover, cancer cells that survive the blockade of ERK1/2 combined with EGFR TKI treatment enter a senescence-like phenotype characterized by high YAP/TEAD activity [18]. The consequences of these situations are complex. First, it appears that mutations in RAS oncogenes cannot occur in the early stages of carcinogenesis if oncogenesis affects cells in which RAS mutations induce senescence. TP53 mutation should precede RAS G12V mutation in the so-called mutational sequence. This phenomenon is observed during the carcinogenesis of colorectal cancer [19]. However, this information also forces us to reflect on therapy. Most efforts are aimed at minimizing the excessive transduction of signals induced by RAS oncogenes, restoring GTPase activity, or preventing interactions between RAS mutants and BRAF protein. However, restoration of TP53 function can sometimes change the molecular context in the cell to one in which RAS mutations lead to senescence (Fig. 2). Senescence itself is of course not as important as apoptosis, but the loss of the ability to proliferate undoubtedly paralyzes tumor progression. The biggest obstacle seems to be the introduction of such a solution in clinical practice. There are no effective small-molecule activators of TP53 mutants, except for the Y220C mutant. To date, there are no conclusive data on whether TP53 Y220C is reactivated in cells with RAS mutations, although research is ongoing [20–24]. On the other hand, gene therapy or in vivo editing of the genome of cancer cells is not possible until the efficiency of the delivery of the therapeutic transgenes or transgenes required for genome editing is significantly greater [25].

It should also be noted that the positive effect on proliferation is not the only pro-oncogenic effect of RAS oncogenes. These additional mechanisms do not appear to undergo such large changes depending on whether TP53 is mutated.

Mutations in genes encoding proteins involved in pathways activated by growth factor receptors have broader consequences, one of which is increased cellular glucose uptake [26, 27]. In normal cells, glucose uptake does not occur without insulin, or a growth factor present in the environment [28]. However, mutations in genes encoding various proteins, including RAS oncogenes, can drive glucose uptake even in the absence of growth factor receptor agonists or insulin.

Another mechanism that has recently been discussed less frequently is the blocking of differentiation under the influence of oncogenes, such as RAS mutants [29]. The existence of cancer stem cells has long been disputed [30, 31]. However, undermining the existence of cancer stem cells does not necessarily translate into undermining the role of oncogenes in blocking differentiation. The fact that many cancers may not have their own cancer stem cells does not change the fact that they can be derived from normal stem cells; the process of cancer cell differentiation may be blocked, and its blockade may have therapeutic effects [32]. In this situation, the blockade of differentiation guaranteed by oncogenes remains very important [33].

Therefore, mechanisms that allow glucose to flow or block differentiation are important. However, in the absence of proliferative ability, inducing senescence after TP53 reactivation may play a positive role. Senescent cells cannot proliferate. Senescent cells increase in size because of glucose uptake; however, this change is far less dangerous than proliferation. It would also be possible to use combination therapy and induce apoptosis, not just senescence, in cells with RAS oncogenes and restored TP53 activity. Naturally, the reactivation of TP53 is likely to cause many different changes in tumor cells, not just the restoration of prosenescence in RAS mutants.

### RAS protein in silico structures

**Fig. 3:**
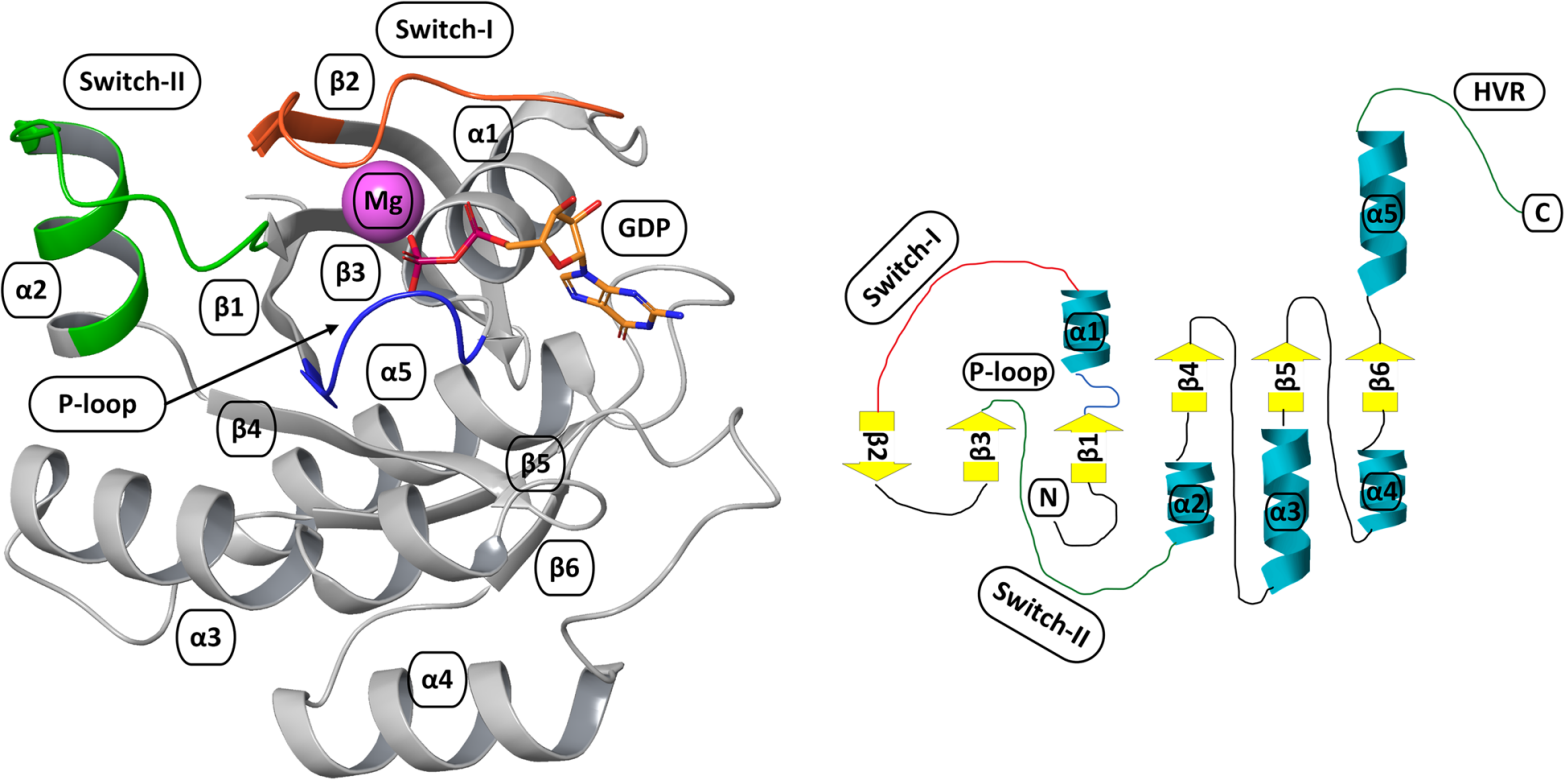
Structural elements of RAS family of proteins. Switch-I and Switch-II – switch regions; α1-α5 – α-helices; β1-β6 – β-sheets; HVR – hypervariable region; P-loop – residues 10-14 of the RAS protein; GDP - guanosine 5′-diphosphate

Despite their relatively small size and number of structural elements, RAS proteins are difficult and elusive drug targets. The four isoforms (NRAS, HRAS, KRAS4A, and KRAS4B) encoded by three human RAS genes exhibit high structural similarity. Publicly available structural data for RAS proteins were initially dominated by HRAS. More recently, as structural differences among RAS isoforms have been discovered, these isoforms are no longer considered identical [34]. The G domain of RAS proteins (residues 1 to 166), which is crucial to their biological function, consists of the protein core formed by six beta-strands surrounded by five alpha-helices. The two most important structural elements for RAS signaling and interaction with downstream effectors and RAS regulators (GEFs and GAPs) are the two switch regions designated switch-I (SW-I) and switch-II (SW-II) (Fig. 3). The switch regions differ in their definitions due to their high intrinsic flexibility. Usually, SW-I encompasses residues 30–40, and SW-II encompasses residues 58–72. Mutations involved in cancer are located in the SW-II or P-loop (residues 10–14) at Q61, G12 and G13 [34]. In addition to the G domain, RAS proteins contain a C-terminal structural element termed the hypervariable region (HVR), which is crucial for RAS membrane association [5]. Recent discoveries of two potentially druggable pockets on RAS, namely, the SW-II and SW-I/II pockets, have brought some hope for drug discovery efforts. The SW-I/II pocket is preferred because of efforts aimed at blocking RAS interactions with regulatory or effector proteins, whereas the SW-II pocket is preferred for locking RAS in an inactive (GDP-bound) conformation [35].

**Fig. 4:**
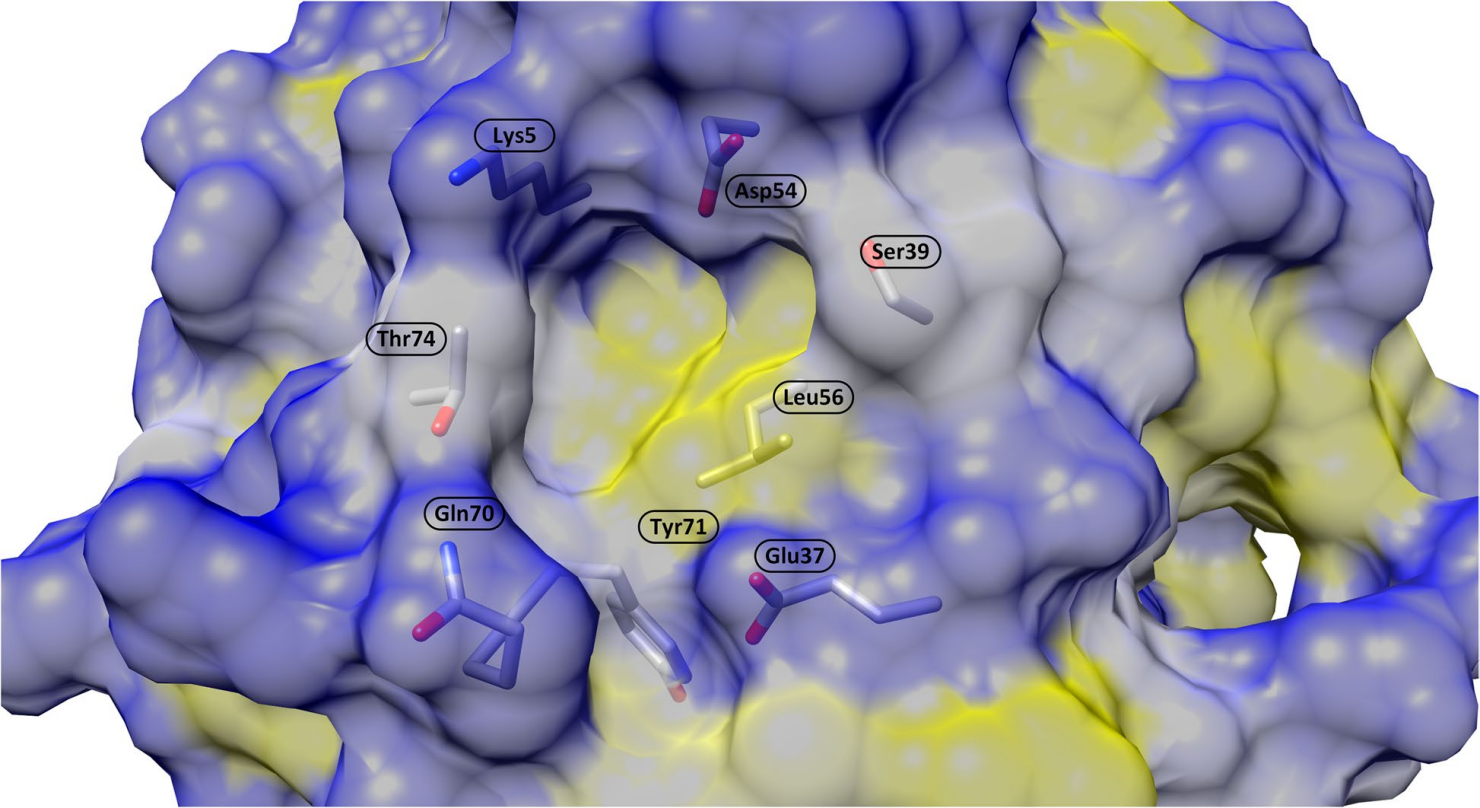
Surface representation of the SW-II pocket around the covalent inhibitor sotorasib (AMG510) based on the PDB structure 6OIM. The surface color corresponds to the Kyte-Doolittle hydrophobicity of the underlying amino acids (ranging from blue 4.5 to yellow -4.5)

The SW-II pocket is an allosteric cavity located between the α2-helices (switch-II) and the α3-helices and the central β-sheet of the RAS (Fig. 4). It includes hydrophobic subpockets and extensions occupied by water molecules, which can provide additional space for growth of inhibitors [36]. The SW-II pocket, discovered by linking covalent fragments [36, 37], can be observed only in inactive (GDP-bound) RAS and is closed in the active (GTP-bound) conformation. Covalent inhibitors of the SW-II pocket have been approved for clinical trials in humans, and recently, the Food and Drug Administration (FDA) has approved sotorasib [38] and adagrasib [39] for the treatment of advanced non-small cell lung cancer (NSCLC) patients harboring the KRAS G12C mutation. Unfortunately, adaptive resistance mechanisms have already been characterized [40]. Secondary mutations acquired by KRAS G12C (e.g., K16T, R68S, Y96C, or Y96D) have been suggested as direct sources of resistance to known small-molecule inhibitors. Computational studies of four KRAS G12C variants resistant to sotorasib (AMG510) and adagrasib (MRTX849) have revealed a detailed molecular resistance mechanism. Studies involving long molecular dynamics (MD) simulations followed by Markov state model (OM) and dynamic network analysis have shown the direct effect of secondary mutations on the conformational transformation of both switch domains, which may affect binding and recognition by downstream effectors [41]. Another resistance mechanism, characterized as a rapid nonuniform adaptive process enabling some groups of cells to bypass the effect of treatment, has been discovered at the single-cell level. The mechanism is based on the expression of the new KRAS G12C allele, which is maintained in a drug-insensitive state by epidermal growth factor receptor (EGFR) and Aurora kinase A (AURKA) signaling [40]. Additionally, targeting the SW-II pocket (observed only in the inactive RAS) is unlikely to be successful on GTP-bound KRAS (e.g., KRAS G12R) [42].

**Fig. 5:**
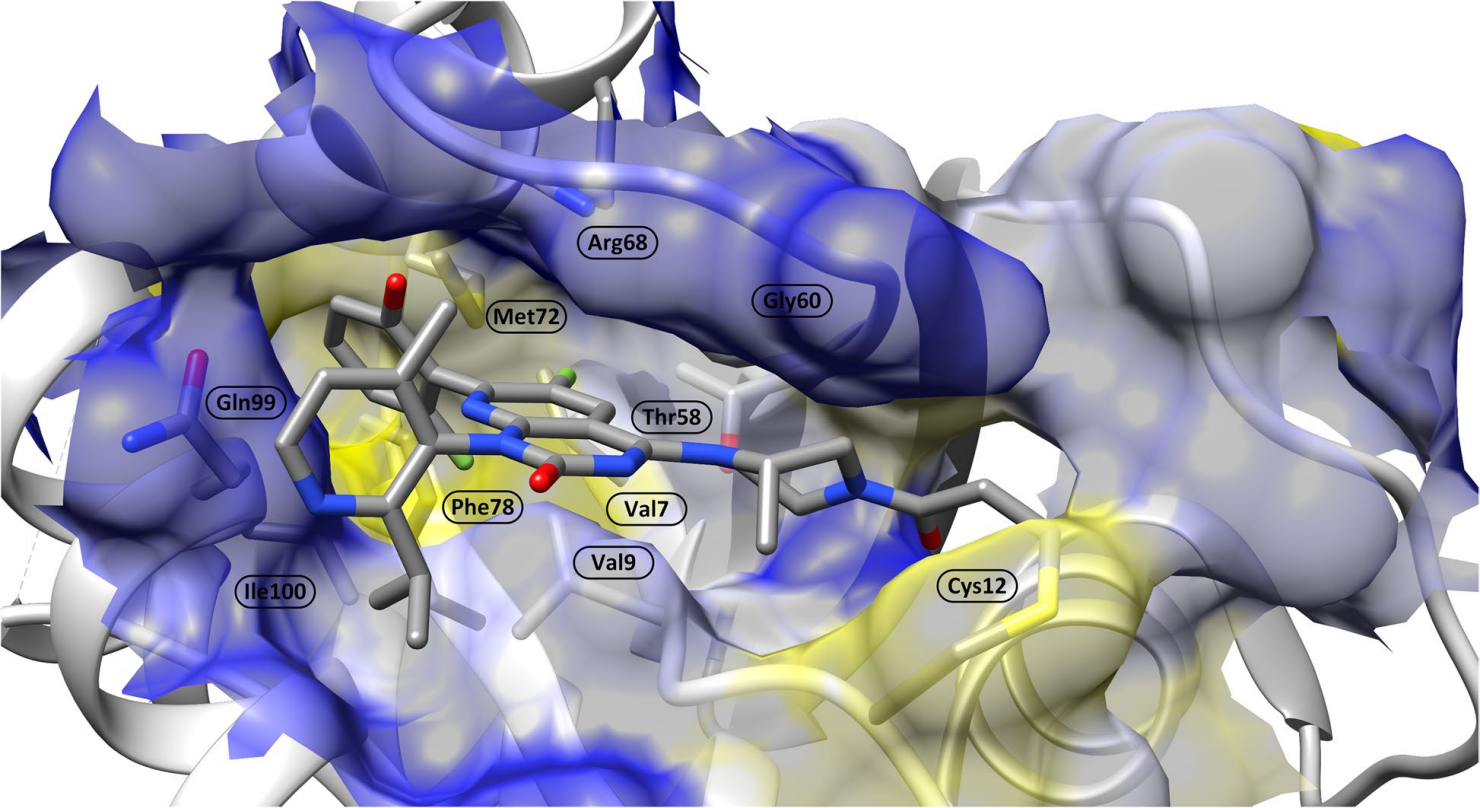
Surface representation of the SW-I/II pocket based on the PDB structure 3OIW. The surface color corresponds to the Kyte-Doolittle hydrophobicity of the underlying amino acids (ranging from blue 4.5 to yellow -4.5)

The less explored SW-I/II pocket is located between the switch regions. The pocket discovered by fragment linking [43, 44] is rather small and shallow. It consists of a small lipophilic region (formed by the K5, V7, D54, L56, Y71, and T74 amino acids) flanked by a shallow polar rim [45] (Fig. 5). The SW-I/II pocket has recently been proposed as a universal target for all RAS isoforms, and the first chemical probe, BI-2852, which binds the pocket in both active and inactive RAS states with nanomolar activity, was proposed [45]. Although indoles have been identified as privileged scaffolds, determining the selectivity between KRAS, HRAS, and NRAS isoforms is highly challenging because all the isoforms have identical sequences in the SW-I/II pocket, and sparing at least one wild-type RAS isoform [45] would be beneficial to minimize toxicity and maintain normal cellular functions [46]. Meanwhile, multi-RAS inhibitors such as RMC-7977 [47] and BI 3706674 (NCT06056024) [48] are designed to target both mutant and wild-type RAS proteins. Preclinical studies have demonstrated significant anti-tumor activity at well-tolerated doses, suggesting a therapeutic window in which cancer cells are preferentially affected over normal cells.

### Small-molecule targeted inhibitors against RAS mutants

Although KRAS mutants have long been considered “undruggable”, many research teams have been working to restore the GTPase activity of mutated KRAS proteins. As previously mentioned, the most common RAS mutations occur at codons 12, 13, and 61, with codon 12 being the most frequently mutated across all three RAS isoforms [49].

KRAS G12C is targetable due to the presence of cysteine residues. The appearance of cysteine instead of glycine leads to the formation of a characteristic spot in the mutated protein and, therefore, provides an opportunity to specifically target altered KRAS [36]. Additionally, KRAS G12C can hydrolyze GTP almost equally as the wild-type form of KRAS, enabling the interaction between KRAS G12C and its downstream effectors [50]. This KRAS G12C feature was used to block the mutant from being inactive by using a small chemical compound, the GDP analog SML-8-73-1. This compound can covalently bind to cysteine 12 of KRAS G12C [51]. SML-8-73-1 was found to be cell impermeable, and attempts were made to deliver it as a caged compound allowing the inhibitor to enter the cell, but also to dramatically decrease its selectivity/specificity and antitumor activity [50]. Therefore, the use of SML-8-73-1 as a drug is unlikely in cancer patients.

After testing several chemical compounds capable of inhibiting KRAS mutants, researchers have developed a compound named ARS-1620. ARS-1620 binds specifically to KRAS G12C mutants, and in vivo studies have shown that ARS-1620 can significantly inhibit tumor growth and regression [52]. Interestingly, the tested molecule exhibited greater effectiveness in 3D in vitro cultures than in 2D cultures [52]. ARS-1620 induced more significant tumor growth inhibition and regression in patient-derived xenografts (PDXs) harboring the KRAS G12C mutation than in other KRAS-mutated PDXs [52].

Substitution of cysteine instead of glycine also provides another opportunity to target the KRAS G12C cysteine residue, and its thiol group can bind to potential small chemical inhibitors through disulfide bridges [53]. This alteration is located near the switch regions involved in GTP binding and interactions with downstream effectors. Therefore, this feature should increase the selectivity and activity of the applied inhibitor.

Sotorasib is the first small molecule inhibitor that directly and specifically targets the KRAS G12C mutant and was introduced into clinical studies. AMG510 binds to Cys12 of KRAS G12C and locks it in its inactive state. Sotorasib was the first FDA-approved drug for patients with non-small cell lung cancer (NSCLC) characterized by the presence of the KRAS G12C mutation, if they received or underwent prior systemic therapy (NCT03600883) [38]. The promising results of these clinical trials suggest that AMG510 is an effective drug for treating KRAS G12C NSCLC patients, and that possible combinations of sotorasib with other anticancer drugs should be tested to determine their safety [54].

The results of the CodeBreaK 200 trial showed that the treatment of NSCLC patients with sotorasib improved progression-free survival (PFS) compared to treatment with docetaxel [55]. Despite promising results, the FDA raised concerns about potential systemic bias, rendering the phase 3 PFS data unreliable for interpretation [56]. A phase Ia/Ib clinical trial testing the combination of sotorasib with the Aurora A kinase VIC-1911 is being conducted in patients with locally advanced or metastatic NSCLC harboring the KRAS G12C mutation (NCT05374538).

Adagrasib (MRTX849) also targets the KRAS G12Cprotein by irreversibly binding cysteine 12 in switch II pocket to lock the molecule in its GDP-bound form leading to the disruption of Ras/MAPK pathway [57]. This leads to the inhibition of MAPK signaling through covalent bonds within the switch-II pocket region, where GTP binds to KRAS protein [58]. In vitro studies have shown that MRTX849 can reduce cell viability even at low concentrations, while in vivo studies have shown that it has a strong dose-dependent inhibitory effect on pERK and, as a result, tumor regression [58]. Like AMG510, ongoing clinical trials have demonstrated the effectiveness and safety of MRTX849 in NSCLC patients with the KRAS G12C mutation. Based on the results of the Krystal-1 phase I clinical trial in which adagrasib showed an objective response in more than 40% of patients with locally advanced or metastatic NSCLC, adagrasib became a second FDA-approved drug for the treatment of KRAS G12C-mutated NSCLC [39]. 

ASP2453 is one of the more recently developed KRAS G12C inhibitors. It selectively binds to the Cys12 residue of the mutated KRAS protein and, as a result, can inhibit tumor growth, KRAS activation and downstream signaling at nanomolar concentrations [59]. ASP2453 also demonstrated faster binding kinetics for the KRAS G12C mutant. ASP2453 has more inhibitory potential than AMG510 and can induce tumor regression in xenograft models resistant to AMG510 [59]. ASP2453 had better results than sotorasib and adagrasib in vivo. Lower doses of ASP2453 induced more significant tumor regression than increasing sotorasib doses in mice with xenografts; however, doses of ASP2453 were more than 3-fold lower than the dose of adagrasib that led to almost complete tumor regression [58, 59]. These results suggest that ASP2453 can be more effective than FDA-approved sotorasib and adagrasib for the treatment of KRAS G12C-positive tumors.

There are several other inhibitors of KRAS G12C that are currently being studied in early phase clinical trials. In a phase I study of patients with previously treated NSCLC, colorectal cancer (CRC), or solid tumors harboring the G12C mutation, treatment with divarasib (GDC-6036) resulted in a clinical response in almost 60% of the patients with NSCLC and a median progression-free survival (mPFS) of 13.7 months. The response rate observed in patients with CRC was nearly 36%, and the median PFS was 6.9 months. The observed treatment-emergent adverse events (TRAEs) were mostly low-grade and manageable with supportive medications and dose modifications, suggesting that divarasib is clinically safe. Divarasib seems to demonstrate more favorable responses and extended progression-free survival rates in individuals diagnosed with either NSCLC or CRC than these reported with current single-agent KRAS G12C inhibitors (NCT04449874) [60]. Krascendo-170 Lung (NCT05789082) is another ongoing phase Ib/II study evaluating the safety and activity of the combination of divarasib and pembrolizumab with other anticancer therapies in patients with previously untreated, advanced or metastatic KRAS G12C-positive NSCLC [61].

Another selective, covalent, and irreversible KRAS G12C inhibitor, JDQ443, is under investigation in a phase Ib/II trial (KontRASt-01; NCT04699188) as monotherapy or in combination with TNO155 (SHP2 inhibitor) and/or tislelizumab (anti–PD-1 monoclonal antibody) in KRAS G12C-mutated solid tumors, including non-small cell lung cancer. Preliminary data on JDQ443 alone showed an acceptable safety profile and clinical activity in an initial cohort of NSCLC patients [62]. When combined with TNO155, JDQ443 demonstrated promising antitumor activity and safety, showing a response in 33.3% of previously treated NSCLC patients, with a 66.7% objective response rate (ORR) and 66.7% disease control rate (DCR) [63]. 

In vitro analyses of cell lines expressing KRAS G12C demonstrated that olomorasib (LY3537982) had tenfold greater inhibitory activity than AMG510 and MRTX849. In vivo studies have demonstrated that LY3537982 monotherapy led to tumor growth inhibition and induced tumor regression when used in combination with other KRAS G12C-targeting drugs [64]. In 2021, the safety, tolerability, and preliminary efficacy of LOXO-RAS-20001 were evaluated in a phase I/II clinical trial involving patients with KRAS G12C-mutated solid tumors (NCT04956640). LY3537982 has proved tolerance in patients who were previously intolerant to other KRAS G12C inhibitors and lacked high-grade liver toxicity when it was used in patients with multiple tumor types [65]. Improved preliminary efficacy was observed when olomorasib was combined with cetuximab, with ORR and DCR reaching 45% and 92%, respectively, and a higher progression-free survival rate than olomorasib alone (7.6 months vs. 4.2 months) in CRC. In addition, this combination exhibited a favorable safety profile, with treatment-emergent adverse events (TEAEs) accounting for 30% or less [66]. In KRAS G12C-mutant advanced NSCLC patients with first-line metastatic disease, regardless of PD-L1 expression, combining LY3537982 with pembrolizumab demonstrated promising antitumor activity and favorable safety, with infrequent low-grade TRAEs [67]. International clinical trials investigating this combination in first-line metastatic NSCLC have initiated enrollment (SUNRAY-01, NCT06119581).

D-1553 (garsorasib) is another potential drug for the treatment of KRAS G12C mutants. In preclinical studies, compared with WT KRAS, KRAS G12C was able to selectively bind and inhibit ERK phosphorylation in cell lines harboring KRAS G12C [68]. D-1553 induced significant tumor regression and led to tumor growth inhibition when used in combination with chemotherapy [68]. D-1553 is currently being tested in a phase I/II clinical trial to assess its safety, tolerability, and pharmacokinetics in patients with advanced or metastatic KRAS G12C-mutated tumors (NCT04585035).

There are also specific RAS(ON) inhibitors such as RMC-6291, a covalent tri-complex inhibitor designed to target the active state of KRAS G12C. This compound binds to cyclophilin A (CYPA) and remodels its surface to create a binary complex for active KRAS, disrupting KRAS-effector interactions and downstream signaling. RMC-6291 in a phase I clinical study (NCT06128551) showed promising antitumor activity in KRAS G12C-mutated solid tumors even with recent prior (OFF) inhibitor treatment, as well as favorable safety with infrequent and manageable treatment-related adverse events [69]. 

RMC-6236 is another investigational drug candidate proposed by Revolution Medicines. Unlike RMC-6291, RMC-6236 is a noncovalent, multiselective inhibitor that targets a wide range of common RAS mutations. This molecule is currently being evaluated in a phase I/Ib trial in patients with advanced solid tumors harboring any nonsynonymous mutation of RAS at codons 12, 13 and 61 (NCT05379985), and demonstrated an acceptable safety profile, causing non severe low-grade treatment-related toxicities and anti-tumor activity across two tumor types (NSCLC and PDAC) [70]. According to updated data from a phase I trial, RMC-6236 showed compelling anti-tumor activity and a favorable safety profile in a broad population of patients with previously treated PDAC [71]. Attempts have also been made to combine RMC-6236 with chemotherapeutic agents. In the treatment of CRC and/or PDAC patients, 3 types of polytherapy are being tested: RMC-6236 with 5-fluorouracil-based regimens; RMC-6236 and cetuximab (anti-EGFR) with or w/o mFOLFOX6, RMC-6236 combined with both gemcitabine and nab-paclitaxel (NCT06445062). The safety and effectiveness of both RMC-6236 and RMC-6291 KRAS inhibitors are evaluated in a phase Ib/II clinical trial in combination with pembrolizumab (anti-PD-1 antibody) and optionally with chemotherapeutics in NSCLC and other KRAS G12C-positive tumors, respectively (NCT06162221).

Novel potential best-in-class therapy designed to effectively block both GTP- (ON) and GDP-bound (OFF) forms of KRAS G12C is under development by Frontier Medicines. FMC-375 is an irreversible covalent dual inhibitor currently being evaluated in a phase I/II PROSPER study of patients with locally advanced unresectable or metastatic solid tumors (NCT06244771). Nonclinical studies demonstrated that FMC-376 results in tumor regression in multiple mouse models including PDX models of NSCLC, PDAC and CR [72–74], suggesting its ability to overcome resistance to KRAS G12C (OFF) inhibitors.

Several drugs with KRAS G12C inhibitory potential, such as LY3499446 and JNJ-74699157, have been found to be ineffective in preclinical studies because of their unfavorable safety profile, and clinical studies need to be terminated (NCT04165031) [75].

Although, there is plenty of approaches at targeting KRAS G12C, KRAS G12D is the most frequently mutated KRAS subtype in solid tumors and remains “undruggable” in clinical settings [76, 77]. Unlike the KRAS G12C mutation, G12D poses significant challenges for the development of covalent inhibitors due to the absence of a reactive residue near the switch-II-binding pocket, making it difficult to apply similar covalent strategies for inhibition [78]. Ongoing research is focused on innovative approaches to overcome these obstacles and improve outcomes in patients with KRAS G12D-driven cancer.

The first groundbreaking small-molecule inhibitor specifically designed to target the KRAS G12D mutation was MRTX1133. It exhibited dose-dependent inhibition of KRAS-mediated signal transduction and marked tumor regression in a subset of KRAS G12D-mutant cell-line-derived and patient-derived xenograft models [79], PDAC in particular [80]. The promising results of MRTX1133 in preclinical studies have prompted its exploration on human subjects. MRTX1133 obtained clearance as an investigational new drug (IND) by the FDA in October 2021 for a phase I/II clinical trial (NCT05737706), targeting patients with advanced solid tumors (PDAC, NSCLC, colorectal cancer, and others) harboring the KRAS G12D mutation [81]. MRTX1133 also synergizes with the next generation nuclear transport protein inhibitor eltanexor resulting in enhanced antitumor activity against PDAC [80].

HRS-4642 is another selective and non-covalent KRAS G12D inhibitor exhibiting anti-tumor efficacy confirmed in preclinical studies, which also synergizes with proteasome inhibitors [82]. The first in human phase I study (NCT05533463) has demonstrated its tolerable safety profile and promising anti-tumor activity in the escalating dosing cohorts [83]. HRS-4642 either as a single agent or in combination with carfilzomib transformed the tumor microenvironment into an immune-permissive state [82].

Another small-molecule inhibitor, QTX3034, is a promising therapeutic candidate targeting KRAS G12D. In preclinical studies it demonstrated good oral bioavailability and promising anti-tumor activity [84]. QTX3034 is currently in IND-enabling development to permit clinical evaluation of single agent and its combination with cetuximab in a phase I trial (NCT06428500) [85].

Novel potential therapy designed to effectively target both GTP- (ON) and GDP-bound (OFF) states of KRAS G12D is under development by GenFlet Therapeutics, in collaboration with Verastem Oncology. GFH375 (VS-7375) demonstrated favorable oral bioavailability and potent efficacy in multiple KRAS G12D tumor models in preclinical studies. It also appeared to be effective in an intracranial tumor model and showed strong synergy with avutometinib (an inhibitor of Ras-Raf-MEK-ERK signaling) [86]. Recently, GenFleet received IND Approval from China’s National Medical Products Administration (NMPA) for GFH375 (VS-7375) in a phase I/II study targeting advanced solid tumor patients with KRAS G12D mutation (NCT06500676) [87].

INCB161734, another KRAS G12D(ON/OFF) inhibitor, demonstrated excellent oral bioavailability and potential efficacy in multiple types of G12D-mutated tumor xenograft models, resulting in significant tumor growth inhibition, growth arrest, and/or regression in multiple PDAC and CRC mouse tumor models. The potential benefit of INCB161734 for patients with KRAS G12D-mutated cancers is under investigation in an ongoing phase I clinical trial (NCT06179160) [88], having a 70% phase transition success rate (PTSR) into phase II [89].

Recently, preclinical studies on TSN1611, a potential KRAS G12D(ON/OFF) inhibitor developed by Tyligand Bioscience, showed its favorable safety profiles and significant in vitro and in vivo anti-tumor activity in various KRAS G12D-mutant models, as well as its brain penetration potential [90]. These findings support further development, and a phase I/II study of TSN1611 in patients with advanced solid tumors harboring the KRAS G12D mutation is currently ongoing (NCT06385925) [91].

Attempts have been made to use RAS(ON) inhibitors for the treatment of KRAS G12D-positive cancers. An example is a selective and covalent KRAS G12D inhibitor RMC-9805. Upon binding to the mutant, RMC-9805 forms a stable complex with KRAS G12D and cyclophilin A, leading to suppression of RAS pathway activity, inhibition of cancer cell proliferation, and apoptosis induction in vitro and in most preclinical PDAC and NSCLC cancer models when used alone [92]. Moreover, the use of RMC-9805 in combination in combination with SHP2 inhibitors resulted in regression of CRC models that were not as responsive to monotherapy. The promising preclinical data resulted in RMC-9805 being currently in IND-enabling development to permit clinical evaluation of single agent and combination strategies in patients with KRAS G12D tumors (NCT06040541) [93].

Not all cancer cells with KRAS mutations require KRAS activation to maintain their viability, which is strongly associated with intrinsic resistance to KRAS G12C inhibitors [94].

### On- and off-target resistance to RAS inhibitors

Clinical trials have shown that KRAS G12C inhibitors may play a crucial role in treating cancer patients harboring the KRAS G12C mutation. Nevertheless, in the case of sotorasib and adagrasib, 50% of patients do not experience significant tumor size reduction, whereas approximately 10% of patients may experience primary disease progression. Unfortunately, cancer patients who experience an objective response or stable disease may progress after treatment with KRAS G12C inhibitors. This process is known as the acquired resistance [94].

Observations made during the testing of potential KRAS G12C inhibitors suggest that there are two main mechanisms of resistance: on-target resistance, which is associated with mutations or amplifications of the KRAS gene; and off-target resistance, which occurs when KRAS is inhibited; however, “bypass” mechanisms occur owing to oncogenic signaling enabled by another effector [95, 96]. Other mechanisms of resistance include mutations implying histological transformation and other alterations that affect apoptosis and/or epigenetic modifications [97].

Primary resistance to KRAS G12C inhibitors relies on KRAS being a component of the RAS-RAF-ERK pathway, which is crucial for cell survival and can stimulate signaling in a KRAS-independent manner. Therefore, despite the use of potentially effective KRAS G12Cinhibitors, cancer cells may maintain their viability [97].

**Fig. 6:**
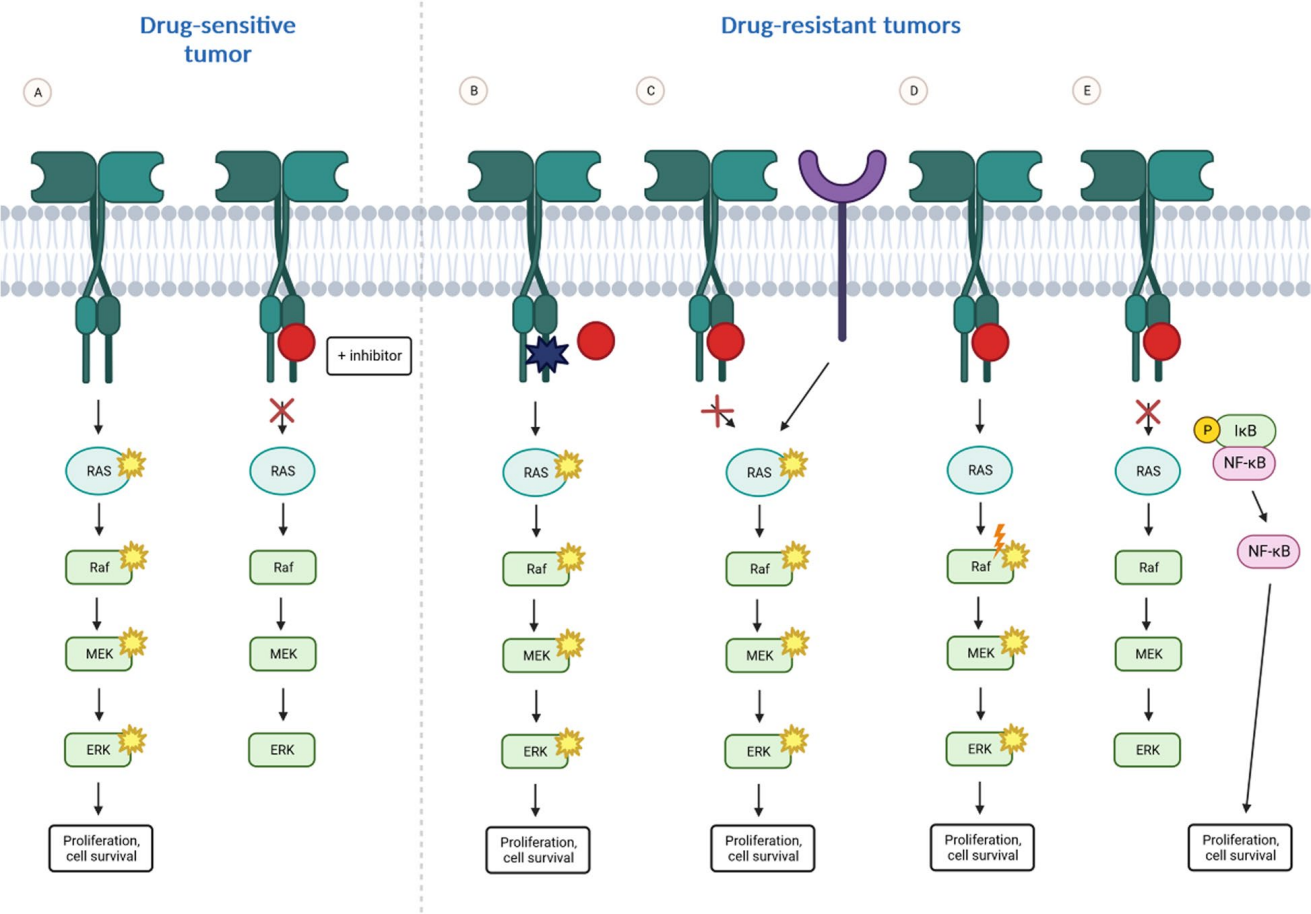
Intrinsic or acquired resistance to TKIs in RAS-mutated tumors in lung cancer, based on [108]

AKT and ERK may be activated even after suppression of the KRAS mutant [98]. This action is possible because of the activation of alternative pathways, such as the PI3K-AKT pathway, due to mutations in PI3KA, AKT1, AKT2, or PTEN. Studies of cell lines with KRAS G12C mutations generated from ARS-1610 and AMG510 and their effects on cancer cells have shown that downstream effectors are still activated and that the levels of active KRAS and HRAS wild-type proteins are increased. It seems that cancer cells can overcome KRAS mutant inhibition through wild-type RAS and RAF proteins, leading to the reactivation of receptor tyrosine kinase (RTK). This mechanism is called adaptive feedback resistance [99] (Fig. 6).

Both downstream activation through alternative pathways and upstream activation occurs after inhibition of KRAS G12C, which correlates with the increased expression levels of EGFR, HER2, FGFR, and c-MET observed in cancer cells [99]. Studies conducted by Xue et al. focused on another mechanism that leads to resistance to KRAS G12C inhibitors. Cancer cells harboring the KRAS G12C mutation entered a quiescent state, in which some of the cells died, while others were able to overcome this inhibition through the synthesis of new KRAS G12C proteins that can be rapidly activated through the upstream effectors EGFR and AURKA [40] (Fig. 6).

As the RAS-RAF-ERK pathway is fundamental for cell survival, it has multiple independent mechanisms that allow it to maintain signaling despite being selectively targeted.

#### On-target resistance

Preclinical and clinical studies have shown that KRAS G12C-mutated cells can acquire resistance to KRAS G12C inhibitors by acquiring mutations within the region to which small-molecule inhibitors bind. In preclinical studies, Ba/F3 cells with the KRAS G12C mutation were treated with sotorasib and adagrasib. Almost 90% of resistant clones acquire additional KRAS mutations. Y96D was the most detected mutation, and this alteration impaired the binding of both inhibitors to the KRAS mutant, even when used at high concentrations [100]. Clinical studies have shown similar observations when samples were obtained from patients with NSCLC harboring the KRAS G12C mutation [97].

Not only additional point mutations of the KRAS protein are being observed in case of acquired resistance to KRAS inhibitors. Awad et al. published data from study including data from 17 cancer patients harboring KRAS G12C mutation in which resistance to adagrasib was observed. Genomic analysis of cancer patient samples has indicated that high-level amplification of KRAS G12C allele is also associated with resistance to KRAS G12C inhibitor [97].

#### Off-target resistance

Several studies and outcomes of clinical trials have shown that cancer cells can overcome KRAS mutation inhibition in a KRAS-independent manner.

During preclinical studies, Suzuki and colleagues observed that cells with the KRAS G12C mutation but resistant to sotorasib also exhibited amplification of MET. Researchers have also tried to knock down amplified MET in these cells using siRNA, and cancer cells retained their sensitivity to sotorasib [101], indicating the importance of MET amplification as an off-target resistance to KRAS inhibitors.

Tests on NSCLC cells have demonstrated that the production of fusion proteins is also a resistance mechanism resulting in downstream activation of MAPK pathway signaling even with inhibition of the KRAS protein [102].

KRAS is the most common RAS isoform among NSCLC patients; however, other isoforms have also been identified. Tanaka et al. described a case in which an NSCLC patient resistant to KRAS G12C inhibitors had three different activating mutations in the NRAS protein [103]. These findings indicate that cells may use the polyclonal activation of RAS isoforms to overcome KRAS inhibition.

Activating point mutations and deletions in the MAP2K1 and BRAF genes are observed among NSCLC patients and are effectors of the MAPK pathway. BRAF V600E is a driver mutation observed in approximately 3% of NSCLC patients, and such patients may be treated with a combination of trametinib (a MEK inhibitor) and dabrafenib (a BRAF inhibitor) [104]. Other activating mutations are also found in the effectors of the PIK3-AKT-mTOR pathway [105].

A study published in 2023 by Adachi et al. emphasized the significance of altering gene expression in cancer cells to trigger the epithelial-to-mesenchymal transition (EMT) when KRAS G12C is inhibited. Through extensive in vitro experiments, researchers discovered that KRAS G12C inhibitors activated yes-associated protein (YAP). They also found that YAP activation was linked to the expression of MRAS, a member of the RAS protein family, which leads to the feedback activation of MAPK signaling. This study further indicated that MRAS expression was associated with a mesenchymal phenotype in the analyzed cells, concluding that YAP activation and MRAS expression are linked to EMT and the development of adaptive resistance following KRAS G12C inhibitor treatment [106]. When induced, EMT leads to intrinsic and acquired resistance to KRAS G12C inhibitors. Adachi et al. showed that cells resistant to KRAS G12C inhibitors maintain activated PI3K, which is regulated mainly by the IGFR-IRS1 pathway. As a result of their studies, they established that combination of inhibitors of KRAS G12C, PI3K and SHP2 demonstrated anti-cancer activity in mouse models of acquired resistance to sotorasib (AMG510) [107].

### Inhibitors of farnesylation

Early efforts to inhibit oncogenic RAS focused on disrupting its membrane localization via C-terminal prenylation, essential for effector signaling [109]. Since RAS undergoes farnesylation by farnesyltransferase (FTase), FTase inhibitors (FTIs) were developed. However, while FTIs effectively blocked HRAS, KRAS and NRAS could bypass this inhibition through geranylgeranyltransferase (GGTase), limiting their therapeutic potential [110, 111]. Dual inhibition of FTase and GGTase was explored but was found to be excessively toxic [112].

To effectively transmit signals from extracellular receptors to intracellular pathways, the KRAS protein must be localized to the inner part of the plasma membrane [113], a process facilitated by a series of posttranslational modifications, beginning with farbesylation [114, 115].

**Fig. 7:**
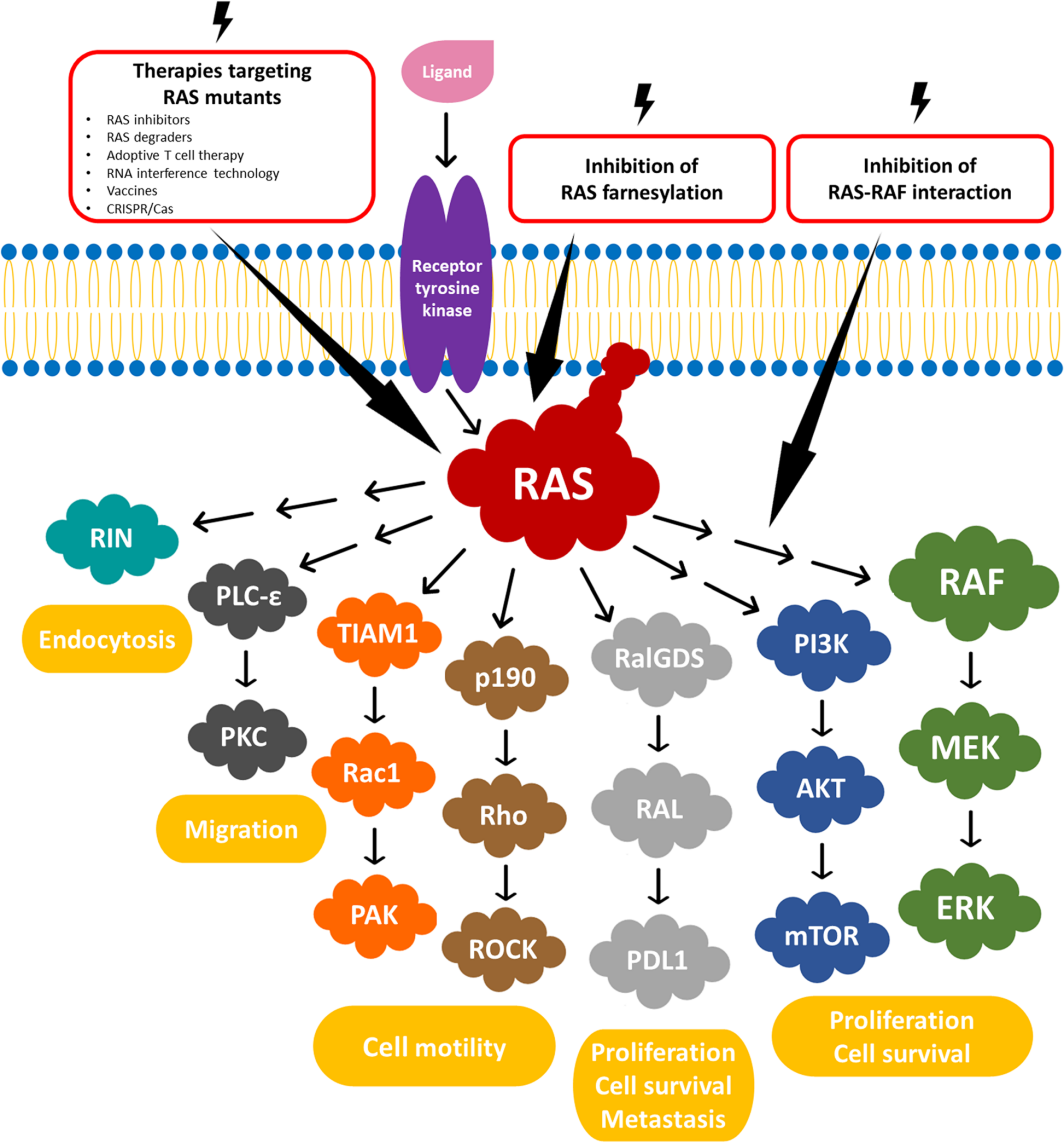
Current approaches to blocking RAS protein based on [114, 149–158]

Since FTase requires the FDP and CAAX motifs of RAS to effectively modify the protein, three classes of FTIs exist: FDP analogs, peptidomimetics of CAAX mimetics, and combinations of both [114] (Fig. 7).

The first identified FTI, α-hydroxyfarnesyl-phosphonic acid [116, 117], was shown to inhibit HRAS modification in vitro [118]. Other FDP analogs also demonstrated selective inhibition of HRAS-driven transformation while sparing normal cells [119]. However, in vivo studies failed to confirm significant anticancer activity, partly due to the challenge of FDP analogs outcompeting natural substrates without inducing systemic toxicity [114]. Efforts to develop non-substrate FTIs led to the creation of tetrapeptides with aromatic substitutions [120]; however, their negative charge limited cell permeability, requiring prodrug modifications [121, 122].

SCH44342, a non-peptidic FTI, inhibited RAS processing with nanomolar potency [123], while Lonafarnib (SCH66336) demonstrated strong selectivity and effectively suppressed tumor growth in KRAS-mutated mouse models [123, 124]. Despite advancing to phase III clinical trials in combination therapies, Lonafarnib's trial was ultimately discontinued(NCT00050336) [124, 125]. Alternative inhibitors like PD083176, which lacks typical cysteine residues and competes with FDP, inhibited FTase in vitro but failed to penetrate cells [114, 119].

The bisubstrate FTI, BMS-186511, demonstrated high specificity, effectively blocking RAS signaling in Hras- and Kras-transformed cells at low concentrations, while sparing normal cells [126–128].

Natural inhibitors, such as manumycin, similarly inhibited KRAS-mutant pancreatic cancer cell lines [129].

Tipifarnib (R115777; Zarnestra), the first non-peptidomimetic FTI tested in clinical trials, demonstrated significant tumor inhibition, although it was only partially effective against KRAS-mutant cells (NCT00006199) [114, 130, 131]. Although the FDA rejected its approval for acute myeloid leukemia (AML), tipifarnib has shown promise in HRAS-mutant head and neck squamous cell carcinoma (HNSCC) (NCT02383927), and in January 2021, the FDA granted a breakthrough therapy designation (BTD) to Zarnestra [132].

L-744832 demonstrated broad anticancer activity in vitro, including in KRAS-mutant cell lines, and was more effective than doxorubicin in tumor shrinkage studies [122]. The drug has entered phase I clinical trials and showed a partial inhibitory effect on FTase, depending on the protein type [133].

Despite setbacks related to toxicity and limited efficacy, FTIs remain a key focus of research. Tipifarnib, in particular, has gained renewed interest as a potential treatment for HRAS-mutant cancers [134] and as part of combination therapy with KRAS G12C inhibitors, such as sotorasib, demonstrating synergistic effects in preclinical lung adenocarcinoma models [135].

### Inhibition of RAS-RAF interaction

Although no effective therapy has been identified for oncological patients diagnosed with RAS mutations, various therapeutic approaches aimed at blocking these mutations are currently being tested in clinical trials. Another treatment strategy for RAS-mutant cancers is to block the interaction between RAS and RAF [136].

The ineffectiveness of previous RAS-targeting small molecules stemmed from the lack of a defined surface pocket for binding. Shima et al. identified binding pockets in the RAS-GTP crystal structure and developed small-molecule inhibitors, Kobe0065 and Kobe2602, which effectively blocked HRAS/RAF interactions, inhibited proliferation, and induced apoptosis in NIH 3T3 cells expressing HRAS G12V. Kobe0065 also demonstrated therapeutic potential in mice implanted with human colon carcinoma SW480 cells expressing KRAS G12V. These results suggest that further investigation of Kobe0065 and its analogs could aid in the development of more effective and safer small-molecule compounds targeting RAS mutants for clinical evaluation [137].

Another therapeutic strategy could involve suppressing the interaction between RAS mutants and effector proteins (including RAF) by targeting the RAS-binding domain (RBD) present in these effectors. Rigosertib (styryl benzyl sulfone, RGB) is an example of a small-molecule compound with this activity. It mimics RAS and binds to the RBD in RAF kinase, preventing the interaction between RAS and RAF. This inhibition disrupts the RAS/RAF/MEK/ERK signaling pathway and suppresses tumor cell proliferation in vitro. Athuluri-Divakar et al. showed that rigosertib inhibits tumor growth in mouse xenograft models of colorectal cancer (KRAS G13D) and lung cancer (KRAS G12S). It may also suppress PI3K/AKT signaling both in vitro and in vivo. Targeting RAS-binding domains responsible for interacting with effector proteins could inhibit RAS signaling pathways and tumor cell proliferation. However, these results require further analysis [138].

Small molecules with protein-protein interaction (PPI) inhibitory activity are also being tested. The iridium(III) compound blocks the interaction between RAS and RAF. The results obtained by Liu et al. indicated that the iridium(III) compound significantly inhibited the interaction of HRAS with RAF-1, signaling progression, MEK signaling, and ERK phosphorylation, both in vitro and in vivo. Iridium(III)induced an increase in the levels of apoptosis markers and promoted growth suppression in A498 (human kidney carcinoma) cell xenografts in BALB/cAnN. Cg-*Foxn1*^*nu*^/CrlNarl mice [139].

Using the bioluminescence resonance energy transfer (BRET) platform, small-molecule blockers that interfere with PPIs (including the RAS-effector complex) were analyzed [140]. Inhibitors derived from intracellular antibody RAS-binding fragments may effectively prevent these interactions. The results of the BRET-based analysis showed that the Abd-7 inhibitor may significantly disrupt the interaction of KRAS and HRAS G12V mutants with CRAF and PI3K in HEK293T cells expressing RAS biosensors. The inhibitor also reduced the viability of human DLD-1 (expressing the KRAS G13D mutant) and HT1080 (with NRAS Q61K mutation) cell lines [141]. Additionally, Abd-8 (known as 3344 compound) inhibited the interactions of different KRAS glycine mutants with CRAF in HEK293T cells transfected with a BRET-based RAS biosensor and decreased the expression of both pMEK and pERK [142].

MCP compounds are another notable example of small-molecule inhibitors of RAS/RAF that have demonstrated activity in both in vitro and in vivo testing phases. González-Pérez et al. showed that MCP110 inhibited RAS/RAF interaction in a *C. elegans* model with constitutive RAS pathway activation, restoring normal vulva development. Additionally, in NIH 3T3 cells with constitutively active RAS, MCP110 disrupted the RAS/RAF signaling pathway [143].

Erbin, a LAP family scaffolding protein, was one of the first identified inhibitors of RAS/RAF interaction. In 2006, Dai et al. suggested that Erbin suppresses RAS/RAF/MEK/ERK signaling by interacting with the Sur-8 scaffold protein, limiting RAF activation [144]. Later studies linked Erbin loss to colorectal cancer progression, showing reduced Erbin expression in patients. Silencing Erbin increased tumor cell proliferation, epithelial–mesenchymal transition, and invasion in 3D cultures, while in Apc knockout mice, it activated RAS/RAF signaling, promoting tumor growth, and reducing survival [145].

Cyclic peptides can disrupt RAS/RAF interactions. Upadhyaya et al. proved that cyclorasin 9A5 inhibits the RAS/RAF complex in H358 lung cancer cells in a dose-dependent manner. In H1299 lung cancer cells, it reduced phosphorylated MEK and ERK levels and induced apoptosis [146]. However, some reports suggest it may lack RAS selectivity [147].

Many therapeutic approaches for RAS-mutant neoplasms have been tested or are still under investigation, and while progress has been made, effective clinical applications remain limited [148] (Fig. 7 and Table 1).

**Table 1:** Strategies for targeting RAS mutants

**RAS targeting approach**		**Target**	**Studies**	**References**
**Blockers targeting KRAS**				
**Small-molecule compounds**	SML-8-73-1	Binds Cys12 of KRAS G12C	In vitro	[51]
	ARS-1620		In vivo	[52]
	AMG510 (Sotorasib)		Clinical trials	NCT03600883
	MRTX849 (Adagrasib)	Inhibits KRAS G12C protein	Clinical trials	[39]
	LY3537982 (Olomorasib)		Clinical trials	[65]
	ASP2453		In vivo	[59]
	D-1553 (Garsorasib)		Clinical trials	[75]
	GDC-6036 (Divarasib)		Clinical trials	[60]
	JDQ443		Clinical trials	[62, 63]
	RMC-6291		Clinical trials	[69]
	FMC-375		Clinical trials	NCT06244771
	RMC-6236	Inhibits KRAS G12X, G13X, Q61X mutants	Clinical trials	[70, 71]
	MRTX1133	Inhibits KRAS G12Dprotein	Clinical trials	[81]
	HRS-4642		Clinical trials	[82]
	QTX3034		Clinical trials	[85]
	GFH375 (VS-7375)		Clinical trials	[87]
	INCB161734		Clinical trials	[88]
	TSN1611		Clinical trials	[91]
	RMC-9805		Clinical trials	[93]
**Inhibitors of RAS fernasylation**				
**FDP analogs**	FDP α-hydroxyfarnesyl-phosphonic acid	Inhibits FTase	In vitro/In vivo	[119]
**Peptidomimetics of CAAX mimetics**	Tetrapeptides with aromatic amino acid substitution		In vitro	[126–128]
	L-744832		Clinical trials	[133]
**Small-molecule compounds**	SCH44342	Compete with CAAX substrate	In vitro	[123]
	SCH66336 (Lonafarnib)		Clinical trials	[125]
	R115777 (Tipifarnib)	Inhibits FTase	Clinical trials	[130, 159]
**Combination of FDP analogs with CAAX tetrapeptide motifs** **(bisubstrates)**	BMS-186511	Inhibits FTase	In vitro	[126, 127]
**Natural products**	Manumycin	Compete with FDP	In vitro	[129]
**Others**	PD083176	Compete with FDP	In vitro	[119]
**Molecules inhibiting RAS-RAF interaction**				
**Small-molecule compounds**	Kobe0065 Kobe2602	Blocks interaction of HRAS/RAF	In vitro	[137]
	Rigosertib	Blocks the RAS-binding domain	In vitro/In vivo	[138]
	Iridium(III) compound	Inhibits interaction of HRAS with RAF-1	In vitro/In vivo	[139]
	Abd-7	Blocks interaction of KRAS-mutants and HRAS^G12V^ with CRAF	In vitro	[141]
	Abd-8 (3344 compound)	Inhibits interactions of different KRAS glycine mutants with CRAF	In vitro	[142]
	MCP110 compound	Blocks RAS/RAF interaction	In vitro/ In vivo	[143]
**Non-steroidal anti-inflammatory drugs (NSAIDs)**	Sulindac and its derivatives	Blocks RAS/RAF interaction	In vitro	[160]
**Cyclic peptides**	Cyclorasin 9A5	Inhibits RAS/RAF complex	In vitro	[146]
**Proteins**	Erbin	Limits RAF activation	In vitro/In vivo	[144]

### Mutated RAS, a negative predictive biomarker? Current clinical recommendations for RAS mutated patients

As shown above, RAS mutations are mostly detected in pancreatic, colorectal and lung cancers. This oncoprotein, commonly known as an “undruggable” oncoprotein that cannot be pharmacologically targeted, is considered the main cause of therapy resistance and poor prognosis [161]. Importantly, there are different clinical recommendations for RAS-mutated patients, specifically for patients with metastatic tumors, depending on the histological tumor type. These differences arose from the first FDA-approved drugs for advanced, KRAS G12C-mutated NSCLC.

In addition to sotorasib, the first-in-class targeted inhibitor of KRAS G12C, for which the accelerated approval was granted based on data from the phase II CodeBreaK 100 trial (NCT03600883) in May 2021 (FDA issued a new postmarketing requirement for an additional confirmatory study to support full approval that will be completed no later than February 2028) and the European Medicines Agency (EMA; 2022) for adult patients with KRAS G12C-mutated locally advanced or metastatic NSCLC who have received at least 1 prior systemic therapy, and adagrasib, approved by the FDA (2022; not yet approved by the EMA) for locally advanced or metastatic NSCLC patients with KRASG12C, currently, there are no approved therapies targeting the RAS protein directly [162–164].

For metastatic colorectal cancer, the latest (2022) European Society for Medical Oncology (ESMO) Clinical Practice Guideline (CPG) for diagnosis, treatment, and follow-up provides recommendations including testing for KRAS, NRAS exons 2, 3 and 4, and BRAF mutations in all patients at the time of metastatic colorectal cancer (mCRC) diagnosis (I, A) due to the limited benefit of anti-EGFR therapies in such patients [165–168].

In contrast, for metastatic oncogene-addicted lung tumors, testing for MET mutations and amplifications, RET rearrangements, KRAS G12C mutations, and HER2 mutations should be performed (II, A). This is because, although for KRAS G12C-mutated NSCLC, first-line treatment algorithms are recommended by the ESMO CPG for non-oncogene-addicted mNSCLC (III, A), for patients with KRAS G12C-mutated NSCLC who fail prior therapy, sotorasib is recommended.

Adagrasib, which is approved by the FDA but not the EMA, is another KRAS G12C inhibitor recommended for the latter. In the registrational phase II cohort, the objective response rate (ORR) was 43% (95% CI 33.5% to 52.6%) for 112 assessable patients. The duration of response (mDoR) was 8.5 months (95% CI 6.2-13.8 months), and the median progression-free survival (mPFS) was 6.5 months (95% CI 4.7-8.4 months) [163, 169].

Moreover, considering alemtuzumab, an anti-PD-L1 immunotherapy for NSCLC patients, it was shown that in the group of non-oncogene-addicted mNSCLC patients, mutations in STK11 and KEAP1 are associated with a poor prognosis, and exploratory subgroup analysis of clinical trials suggested that they are associated with lower immune checkpoint inhibitor (ICI) efficacy, especially in KRAS-mutated tumors [170–172].

What clinical solutions can be offered to patients with mutations in RAS proteins other than G12C, who do not respond to systemic chemotherapy? The coincidence of RAS protein mutations with other molecular markers appears to be crucial for targeted therapy. However, neither EGFR nor RAS mutations in NSCLC nor BRAF and RAS mutations in CRC frequently cooccur, and they are generally mutually exclusive, suggesting functional redundancy [173]; CRC: [174, 175]; NSCLC: [176–179]. In spite of that, small percentages of cancers in which these changes cooccur have been reported [180, 181].

Nonetheless, it has been shown that KRAS mutations, in addition to EGFR, BRAF and PIK3CA mutations, can act as predictive markers of the response to targeted therapy via the use of EGFR-TKIs in patients with NSCLC [181]. Pao et al. showed that routine testing for EGFR and KRAS mutations should be conducted, and their results should be considered when deciding who should be treated with gefitinib or erlotinib [177]. Tumors for which a significant reduction in mass was observed during treatment with gefitinib or erlotinib (22 in total) were characterized by a normal KRAS gene. Most of these cancers harbored mutations in EGFR. Conversely, tumors with an abnormal *KRAS* gene (nine in total) did not shrink during treatment with gefitinib or erlotinib. Thus, patients with KRAS mutations are unlikely to respond to EGFR tyrosine kinase inhibitor (TKI) therapy. Mutated KRAS status was indicated to be a predictor of resistance to cetuximab therapy, not only in NSCLC but also in colorectal cancer, and was associated with a worse prognosis [182, 183]. These and subsequent studies led to the conclusion that RAS mutation status may be related to who may benefit from strategies targeting EGFR, which is reflected in the latest recommendations for the clinical management of patients. In addition to the abovementioned sotorasib, which was indeed a true breakthrough in the treatment of mNSCLC KRAS G12C-positive patients [184], the latest European Society of Medical Oncology (ESMO) recommendations indicate that no EGFR-targeted therapy (alone or in combination with systemic therapy in numerous clinical trials) has been effective in RAS-mutated patients [165]. ESMO consensus guidelines for the management of patients with metastatic colorectal cancer confirm the negative predictive value of RAS mutations. The same situation occurs when TKI administration is considered in BRAF-mutated patients since the BRAF V600E mutation, together with the KRAS mutation, is a strong negative prognostic factor in mCRC. Therefore, BRAF mutation status should be assessed simultaneously with RAS test results for prognostication [165].

Summarizing the relative values of mutational status for single targeted therapies, although EGFR mutation allows for the prediction of a longer progression-free survival rate [185–189], an adverse prognosis is associated with patients harboring KRAS mutations [190, 191]. From a molecular point of view, these differences may result from either intrinsic resistance that arises before therapy, or acquired resistance induced by treatment. Several cellular mechanisms have been proven to be responsible for such resistance to TKIs (Fig. 7). Among these pathways, constitutive activation of the MAPK signaling pathway is a major cause of resistance to anti-EGFR therapies in KRAS- and BRAF-mutated cancers [192]. Finally, targeting multiple proteins in critical signaling pathways appears to be a promising concept for RAS-mutated patients [108] (Fig. 6).

In parallel with the development of targeted therapies directly targeting mutated RASs, attempts have been made to use inhibitors downstream of the RAS protein, or to combine therapies targeting multiple proteins in signaling pathways in cells with RAS mutations. New directions, such as parallel inhibition of PI3K/AKT and MAPK pathways, should be considered [161, 193]. Among alternative therapeutic options, BRAF inhibitors (vemurafenib or dabrafenib) and MEK1/MEK2 inhibitors (trametinib or cobimetinib) have been used alone or in combination [194]. However, several molecularly targeted therapies are associated with initial optimistic responses, followed by acquired resistance due to mutations or activation of signaling pathways [195, 196]. Interestingly, studies have revealed regulatory feedback upon MEK inhibition via interaction with HER family membrane receptors, such as insulin-like growth factor-receptor 1 (IGF1) and c-MET [197, 198].

To overcome resistance to single-targeted therapies, rational polytherapy, which may be effective in RAS mutation-positive cancer patients, has been proposed and tested. Several clinical trials of combination therapies for RAS-mutated CRC and NSCLC have been conducted. A strong synergy was observed between avutometinib (VS-6766, a small molecule MEK/pRAF inhibitor) and defactinib (VS-6063, a small molecule adenosine 5’-triphosphate (ATP) competitive, reversible FAK inhibitor) in KRAS G12V mutant cell lines, in effect, supporting the phase II study evaluating this drug combination for the treatment of recurrent NSCLC with KRAS G12V or other KRAS mutation (NCT04620330) [199]. In addition to its combination with defactinib, avutometinib has shown synergistic activity with other inhibitors in both in vitro and in vivo solid tumor models. There are currently several recruiting clinical trials for VS-6766 in combination with: adagrasib (in KRAS G12C NSCLC patients; NCT05375994), sotorasib (in KRAS G12C NSCLC patients; NCT05074810) or defactinib with gemcitabine and nab-paclitaxel (in patients with pancreatic cancer; NCT05669482). To date, four MEK inhibitors, trametinib, binimetinib, selumetinib, and cobimetinib, have been approved by the FDA [200–203]. Jänne et al. showed that the MEK inhibitor selumetinib combined with docetaxel had a synergistic effect on advanced KRAS-mutated NSCLC [204]. However, a later randomized, multicenter, placebo-controlled, phase II study of selumetinib combined with docetaxel for KRAS-mutant advanced non-small-cell lung cancer did not reveal a statistically significant improvement [205]. Docetaxel was also combined with the MEK1/MEK2 inhibitor trametinib (GSK1120212) in a randomized phase II study of KRAS-mutant advanced NSCLC [206]. Since KRAS mutations are detected in as much as 25% of NSCLCs, and no targeted therapies have been approved for this subset population, trametinib, a selective allosteric inhibitor of MEK1/MEK2 that has preclinical and clinical activity in KRAS-mutant NSCLC, was analyzed here. Blumenschein et al. reported a phase II trial comparing trametinib with docetaxel in patients with advanced KRAS-mutant NSCLC but concluded that the PFS and response rate of patients with previously treated KRAS-mutant-positive NSCLC were similar to those of patients with docetaxel. In contrast, patients with RAS-mutated CRC treated with selumetinib combined with cetuximab exhibit two partial responses and two long-lasting stabilization regimens [207]. Furthermore, Ziemke et al. verified that cotargeting MEK and CDK4/6 could be effective in treating KRAS-mutant colorectal cancers and was highly effective in all three KRAS-mutant colorectal cancer PDX models [208]. Alternatively, Vallejo et al. considered the inhibition of Fos-like antigen 1 (FOSL1), a downstream transcription factor, to show therapeutic promise in KRAS-mutant lung and pancreatic cancer [161, 209].

Despite recent therapeutic progress, the prognoses of mNSCLC patients (with no KRAS G12C mutation) and mCRC patients with KRAS mutation have remained poor, and further analyses are needed to improve their therapeutic outcomes.

### Novel therapeutic approaches

Since early attempts to directly target RAS or its posttranslational modifications failed [111, 210–212], emerging approaches have focused on upstream and downstream mediators to suppress its oncogenic signaling.

The research conducted by Osterem and Shokat proved to be a turning point in drugging the previously “undruggable” Ras [36]. This breakthrough discovery has led to the development of a series of small molecules, most of which initially showed insufficient therapeutic potential both in vitro and in vivo [36, 51, 213]. Nevertheless, the search for perfect drugs has intensified, leading to the discovery of new generation of molecules that bind directly to the mutated RAS protein, inhibiting its oncogenic activity; these include the abovementioned sotorasib and adagrasib. Other direct inhibitors are currently in early-phase clinical trials or preclinical studies (Table 2). Although mutations such as G12D and G12V are more frequent in most KRAS-positive tumors, research on their selective inhibitors remains in the preclinical phase (Table 2).

**Table 2:** Overview of the novel therapeutic strategies against oncogenic RAS

**Strategy and target**	**Agent**	**Combination drug**	**Condition/Disease**	**Phase of development**	**Trial ID/Reference**
**Mutant specific direct Ras inhibitors**					
***RAS G12C***	Sotorasib (AMG510)	MVASI; anti PD -1/L1; VS-6766	NSCLC	Phase I/II	NCT05180422; NCT03600883; NCT05074810
		RMC-4630		Phase II	NCT04933695; NCT04625647; NCT05398094; NCT04933695; NCT05638295; NCT05054725
		Panitumumab		Phase III	NCT04303780; NCT05198934
	Adagrasib (MRTX849)	Nivolumab; pembrolizumab	NSCLC	Phase II	NCT05162443; NCT05472623; NCT04613596; NCT05609578; NCT04613596
		-		Phase III	NCT04685135
		VS-6766		Phase I/II	NCT05375994
		TNO155	Solid tumors	Phase I/II	NCT04330664; NCT03785249
		Palbociclib	Metastatic Pancreatic Cancer	Phase I	NCT05634525; NCT05178888
		BI 1701963	Solid tumors	Phase I	NCT04975256
		Cetuximab	CRC	Phase III	NCT04793958
		Pembrolizumab, cetuximab, afatinib	Solid tumors	Phase Ib	NCT04613596
	GFH925	-	NSCLC and gastrointestinal tumors	Phase I/II	NCT05005234
	Garsorasib (D-1553)	-	NSCLC	Phase Ib/II	NCT05492045
		-	NSCLC, CRC	Phase I/II	NCT04585035; NCT05383898
	JDQ443		NSCLC	Phase II	NCT05445843
		Trametinib, ribociclib, cetuximab; TNO155, tislelizumab	Solid tumors	Phase Ib/II	NCT05358249; NCT04699188
		-	NSCLC	Phase III	NCT05132075
	BI 1823911	BI 170196	Solid tumors	Phase Ia/Ib	NCT04973163
	JAB-21822	Cetuximab	NSCLC, CRC, Solid tumors, PDAC	Phase I/II	NCT05002270
		JAB-3312		Phase I/IIa	NCT05009329; NCT05288205
		Cetuximab	CRC, Small Intestine Cancer, Appendiceal Cancer, NSCLC	Phase Ib/II	NCT05194995; NCT05276726
	MK-1084	Pembrolizumab	Solid tumors	Phase I	NCT05067283
	Divarasib (GDC-6036)	Atezolizumab, cetuximab, bevacizumab, erlotinib, GDC-1971, inavolisib	NSCLC, CRC, Solid tumors	Phase Ia/Ib	NCT04449874
		-	NSCLC	Phase II/III	NCT03178552
		Pembrolizumab, carboplatin, cisplatin, pemetrexed	Previously untreated, advanced or metastatic NSCLC	Phase Ib/II	NCT05789082
	JNJ-74699157 (ARS-3248)	-	NSCLC, Solid tumors	Phase I	NCT04006301
		Abemaciclib, erlotinib, pembrolizumab, temuterkib, cetuximab, LY3295668, TNO155	Solid tumors	Phase Ia/Ib	NCT04956640
	ARS-1620	-	Unspecified	Preclinical	[52]
	ARS-853	-	Unspecified	Preclinical	[213]
	RM-018	-	Unspecified	Preclinical	[103]
	RMC-6291	-	NSCLC, CRC, PDAC, solid tumors	Phase I/Ib	NCT05462717
		RMC-6236	NSCLC, CRC, PDAC	Phase Ib	NCT06128551
		RMC-6236, pembrolizumab, cisplatin, carboplatin, pemetrexed	NSCLC	Phase Ib/II	NCT06162221
	Olomorasib (LY3537982)	Abemaciclib, erlotinib, pembrolizumab, temuterkib, LY3295668, cetuximab, TNO155	NSCLC, CRC, Endometrial Neoplasms, Ovarian Neoplasms, Pancreatic Neoplasms	Phase Ia/Ib	NCT04956640
	FMC-375	-	Advanced solid tumors, NSCLC, CRC, pancreatic cancer	Phase I/II	NCT06244771
***KRAS G12D***	MRTX1133	-	Unspecified	Preclinical	[236]
		-	PDAC	Preclinical	[78]
		Eltanexor (KPT-8602)	PDAC	Preclinical	[80]
		-	NSCLC, CRC, PDAC	Phase I/II	NCT05737706
	HRS-4642	Carfilzomib	Unspecified	Preclinical	[82]
		-	Solid tumors	Phase I	NCT05533463
	QTX3034	Cetuximab	Advanced solid tumors	Phase I	NCT06428500
	JAB-22000	-	PDAC, CRC, NSCLC	Preclinical	[237]
	RMC-9805 (RMC-036)	-	NSCLC, CRC, PDAC	Phase I	NCT06040541
		-	PDAC, NSCLC	Preclinical	[93]
	GFH375 (VS-7375)	-	Advanced solid tumors	Phase I/II	NCT06500676
	INCB161734	Cetuximab, retifanlimab	Advanced or metastatic solid tumors	Phase I	NCT06179160
	TSN1611	-	Advanced solid tumors	Phase I/II	NCT06385925
	BI-2852	-	Unspecified	Preclinical	[45]
***KRAS G13C***	RMC-8839	-	Unspecified	Preclinical	[238]
***KRAS G12V***	JAB-23000	-	NSCLC	Preclinical	[239, 240]
***KRAS***^***MULTI***^	JAB-23400	-	PDAC, CRC, NSCLC	Preclinical	[241]
**Pan-(K)RAS inhibitors**					
***Pan-RAS***	RSC-1255	-	Lung Cancer, Colon Cancer, Glioblastoma, Pancreatic Cancer	Phase Ia/Ib	NCT04678648
***RAS***^***MULTI***^	RMC-6236	-	NSCLC, CRC, PDAC	Phase I/Ib	NCT05379985
		mFOLFOX6, bevacizumab, mFOLFIRINOX, cetuximab, gemcitabine, nab-paclitaxel	CRC, PDAC	Platform study	NCT06445062
	RMC-7977	-	PDAC	Preclinical	[48]
***Pan-KRAS (G12D/V, KRAS wild-type)***	BI-panKRAS1-4	-	Unspecified	Preclinical	[242]
	BI 3706674	-	Unresectable metastatic gastric, esophageal, and gastroesophageal junction adenocarcinoma	Phase I	NCT06056024
***Pan-KRAS (G12C/D/V/A, G13C, A146T/P, Q61E/P, KRAS wild-type)***	BI-KRASdegrader1	-	Unspecified	Preclinical	[241, 242]
**RAS degraders (PROTACs)**					
***KRAS G12C***	LC-2	-	Unspecified	Preclinical	[150]
	YF135	-	NSCLC, Lung Adenocarcinoma	Preclinical	[243]
***KRASG12C/D/V, Q61H***	K27-SPOP	-	Unspecified	Preclinical	[221]
***KRAS G12D***	ASP3082	-	PDAC, CRC, NSCLC	Preclinical	[222]
		Cetuximab and chemotherapy	Previously treated locally advanced or metastatic solid tumors	Phase I	NCT05382559
	ASP4396	-	Locally advanced or metastatic solid tumors	Phase I	NCT06364696
**RAS toxins**					
***pan-RAS***	RRSP-DT_B_	-	Unspecified	Preclinical	[244]
**Adoptive cell therapy**					
***KRAS G12D***	NT-112 (TCR-T cells)	-	NSCLC, CRC, PDAC	Phase I	NCT06218914
	anti-KRAS G12D mTCR PBL	Cyclophosphamide, fludarabine, aldesleukin	Gastrointestinal Cancer, Pancreatic Cancer, Gastric Cancer, CRC	Phase I/II	NCT03745326
***KRAS G12V***	anti-KRAS G12V mTCR PBL				NCT03190941
	TCR Transduced T cells	Cyclophosphamide, fludarabine, anti-PD-1	Pancreatic Cancer, PDAC	Phase I/II	NCT04146298
***KRAS G12D***	NK cell therapy	-	PDAC	Preclinical	[225]
**Cancer immunotherapy**					
**Cancer vaccines**					
***KRAS G12D/V/C, G13D***	mRNA-5671/V941	Pembrolizumab	NSCLC, Pancreatic Cancer, CRC	Phase I	NCT03948763
***KRAS***^***MULTI***^	mutant KRAS-targeted long peptide vaccine	Nivolumab, ipilimumab	CRC, Pancreatic Cancer	Phase I	NCT05013216; NCT04117087
***KRAS G12D/V/R/C***	mDC3/8-KRAS vaccine	-	PDAC	Phase I	NCT03592888
***KRAS G12D/R***	ELI-002 2P	-	PDAC, CRC, NSCLC, ovarian cancer, cholangiocarcinoma, bile duct cancer, gallbladder carcinoma	Phase I	NCT04853017
***RAS***^***MULTI***^	ELI-002 7P	-	PDAC, CRC	Phase I/II	NCT05726864
	GI-4000	ALT-803, ETBX-011, haNK, avelumab, bevacizumab, capecitabine, cyclophosphamide, fluorouracil, leucovorin, nab-paclitaxel, omega-3-acid ethyl esters, oxaliplatin; ETBX-021 (HER2), ETBX-051 (Brachyury), ETBX-061 (MUC1), GI-6207, GI-6301; N-803, regorafenib; cisplatin, necitumumab	Pancreatic cancer, CRC, SCC, triple negative breast cancer	Phase Ib/II	NCT03329248; NCT03387098; NCT03586869; NCT03136406; NCT03563157; NCT03387111; NCT03387085
**RNAi technologies**					
***various mutant KRAS mRNAs***	siRNA coated with peptide-based nanoparticles	-	Pancreatic cancer	Preclinical	[154]
***KRAS G12D mRNA***	siG12D LODER	-	PDAC, pancretic cancer	Phase I	NCT01188785
		Gemcitabine, nab-paclitaxel, FOLFIRINOX		Phase II	NCT01676259
	iExosomes	-	PDAC, pancreatic cancer	Phase I	NCT03608631
**Gene editing**					
***whole KRAS knockout***	CRISPR/Cas9	-	PDAC	Preclinical	[227]
		-	CRC	Preclinical	[228]
***KRAS G12V/D, G13D***		-	CRC	Preclinical	[151]
***KRAS G12D/C***		-	NSCLC	Preclinical	[245]
***KRAS G12V/D***		-	CRC, pancreatic adenocarcinoma	Preclinical	[229]
***KRAS G12D***		-	Pancreatic cancer	Preclinical	[152, 230]
		-	PDAC	Preclinical	[231]
***KRAS G12S***	CRISPR/SpCas9 and dCas9-KRAB	-	NSCLC	Preclinical	[232]
***whole KRAS knockout***	CRISPR/dCas9/HDAC1	-	NSCLC, CRC	Preclinical	[246]
***KRAS G12D***	CRISPR-Cas13a	-	Pancreatic cancer	Preclinical	[234]
	CRISPR-CasRx	-	PDAC	Preclinical	[233]
***KRAS G12C/S***	CRISPR/Cas12a	-	Lung cancer	Preclinical	[235]

Blocking mutated RAS alone may be insufficient to achieve therapeutic goals due to the presence of resistance mechanisms in the complex signaling cascade. Therefore, combination strategies of direct inhibitors together with RAS protein upstream regulators (e.g., SH2-containing protein tyrosine phosphatase-2 (SHP2) and Son of sevenless homolog 1 (SOS1)) as well as downstream signaling effectors (e.g., RAF, MEK, and ERK) have emerged [94, 214–217]. RNA interference (RNAi) technology constitutes an alternative approach to small molecules directly targeting oncogenic RAS (Fig. 7). 

Small interfering RNAs (siRNAs) are promising antitumor agents due to their precision and broad range of potential therapeutic targets. Unfortunately, since they exhibit a short half-life and susceptibility to intracellular degradation, it is crucial to find a solution that would enable them to avoid this problem. Strand et al., using the pancreatic ductal adenocarcinoma mouse model KPPC (p48-CRE/Lox-stop-Lox(LSL)-Kras^G12D^/p53^flox/flox^), showed that peptide-based nanoparticle-coated siRNA is precisely delivered to the tumor microenvironment (TME) and effectively promotes its regression [154]. Other delivery systems, such as local drug eluteR (LODER) and engineered exosomes (iExosomes), are currently in clinical development (Table 2).

Over the last ten years, intensive research has resulted in the proposal of completely new solutions based on technologies that stimulate the patient’s immune system to fight RAS-driven tumors such as vaccines, adoptive T-cell therapy, proteolysis targeting chimeras (PROTACs), and gene editing systems (clustered regularly interspaced short palindromic repeats (CRISPR)/Cas) (Fig. 7).

There are many ways to induce cancer-specific immune responses, including mRNA-, peptide- and dendritic cell-based vaccines. Several clinical studies are ongoing to evaluate the safety and efficacy of KRAS-targeted vaccines. V941 (mRNA-5671), a lipid nanoparticle-formulated mRNA vaccine targeting the most common mutations (G12D, G12V, G12C and G13D), is currently being tested with or without pembrolizumab (an anti-PD-1 antibody) in a phase I clinical trial in patients with advanced or metastatic non-small cell lung cancer, colorectal cancer, or pancreatic adenocarcinoma (NCT03948763; Table 2). A phase I clinical trial of the mutant KRAS-targeted long peptide vaccine alone (NCT05013216; Table 2) or in combination with nivolumab (PD-1 inhibitor) and ipilimumab (anti-CTLA-4 antibody) (NCT04117087; Table 2) are also conducted. mDC3/8-KRAS is an example of a vaccine based on autologous mature dendritic cells pulsed with specific mutant KRAS peptides derived from PDAC patients, who are currently being recruited for phase I clinical trials (NCT03592888; Table 2). First-in-human phase I trial of ELI-002 2P (Elicio Therapeutics, Inc.) focused on its ability to treat minimal residual disease (MRD) in patients with KRAS/NRAS-mutated solid tumors (NCT04853017; Table 2). This immunotherapy comprises an amphiphile (Amph)-modified G12D and G12R mutant KRAS peptides together with an Amph-modified CpG oligonucleotide adjuvant designed to expand polyfunctional mutant KRAS-specific T cells [153]. The ELI-002 7P vaccine is a broad-spectrum vaccine comprising lymph node-targeted (Amph)-modified G12D, V, R, C, S, A, and G13D mutant KRAS peptides. It has been demonstrated that ELI-002 7P has better safety profile than the prior formulation and shows preliminary antitumor efficacy. The randomized phase II is now open in patients with PDAC (AMPLIFY-201; NCT05726864) [218, 219].

PROTAC technology involves the elimination of a protein of interest (POI) by degradation through the ubiquitin-proteasomal enzyme system [220]. Bond et al. recently discovered the first reversible covalent PROTAC, LC-2, capable of degrading endogenous KRAS G12C by combining MRTX849with the von Hippel-Lindau (VHL) E3 ligase ligand, inducing its degradation [150]. Another investigated biodegrader, K27-SPOP, induced ubiquitination of G12C, G12D, Q61H, and G12V, as well as wild-type KRAS in vitro [221]. The novel and advanced KRAS G12D degraders (PROTACs) in development are ASP3082 and ASP4396. Preclinical studies showed that ASP3082 induced degradation of KRAS G12D protein, inhibition of KRAS downstream proteins, and an apoptotic response, and exhibited potent dose-dependent antitumor activities in multiple KRAS G12D-mutated cancer models (PDAC, CRC, and NSCLC) [222]. A phase I study of ASP3082 in patients with previously treated advanced solid tumors and KRAS G12D mutations is currently ongoing (NCT05382559) [223]. ASP4396 is currently in a phase I trial in patients with locally advanced (unresectable) or metastatic solid tumors harboring KRAS G12D mutation (NCT06364696), with a phase transition success rate (PTSR) of 70% for progression to phase II [224].

Adoptive T-cell therapies (ATCs), involving the administration of peripheral blood lymphocytes transduced with a murine T-cell receptor (mTCR) recognizing the G12D and G12V variants of mutated RAS in patients with HLA-A*11:01-expressing pancreatic and gastrointestinal cancers, have entered phase I clinical trials to determine their safety and efficacy in tumor regression (NCT03745326, NCT03190941; Table 2). Recently, Hu et al. proposed NK cell-based adoptive transfer immunotherapy for PDAC. They evaluated the therapeutic potential of transferred NK cells against tumors harboring the G12D KRAS mutation in KPC mice and discovered their significant influence on delaying tumor growth [225].

NT-112 by Neogene Therapeutics is an autologo us T-cell therapy designed to specifically target the KRAS G12D mutationpresented by the HLA-C*08:02 allele. The T cells in NT-112 are genetically engineered to include disruption of the gene encoding transforming growth factor beta receptor type 2 (TGFBR2) to reduce the immunosuppressive effect of TGF-β in the tumor microenvironment and is currently being investigated in a phase I clinical trial in patients with unresectable, advanced, and/or metastatic KRAS G12D-driven solid tumors (NCT06218914) [226].

CRISPR-based gene editing technology is currently in the spotlight due to promising data obtained from preclinical studies on its potential as a therapeutic tool. One of the most used systems for treating RAS-driven cancers is the CRISPR/Cas9 system, which results in gene depletion. In 2017, Muzumdar et al. attempted to use CRISPR/Cas9 in PDAC therapy utilizing both in vitro and in vivo models to target whole mutated KRAS [227]. Wan et al., in turn, proved that complete endogenous KRAS ablation is a promising approach for treating patients with CRC [228]. Mutant KRAS alleles, including G12V/D/C, G13D [151, 152, 229–231], and G12S [232], have also been targeted by the CRISPR/Cas9 system to evaluate their therapeutic potential in PDAC, NSCLC, CRC cells, and animal models. The G12D mutation in KRAS has also been studied using the CRISPR/CasRx system. Jiang et al. indicated that CasRx precisely and effectively silenced the G12D mutant of KRAS in PDAC cells. Moreover, the application of this system to an orthotopic PDAC model resulted in the suppression of tumor growth and improved survival in mice [233]. Other systems tested in preclinical studies include CRISPR/Cas13a and Cas12a. The former has been used to evaluate the therapeutic potential in KRAS G12D-driven PDAC both in vitro and in vivo [234], whereas the latter has been found to be a promising tool for developing therapies to treat lung cancer harboring G12C and G12S mutations. However, these outcomes require further investigation due to the small sample size [235].

## Conclusions

The intricate role of the RAS signaling pathway in tumorigenesis highlights the need for a thorough understanding of RAS biology to develop more effective therapeutic approaches. More than three decades of intense research and efforts to target RAS-driven cancers have led to the development of both clinical and preclinical inhibitors. Notably, the FDA approval of allele-specific KRAS G12C inhibitors has marked a significant milestone in the therapeutic strategies to target RAS-mutant cancers. Numerous alternative strategies are currently being explored, including the development of inhibitors specifically designed for distinct RAS-mutant subtypes. Novel inhibitors, such as LY3537982 and GDC-6036, show considerable promise; however, achieving effective and selective RAS inhibition remains a major challenge due to the emergence of diverse resistance mechanisms. Furthermore, while the RAS oncogene has long been regarded as primarily pro-tumorigenic, recent evidence suggests a more nuanced role in oncogenesis, including context-dependent effects such as the induction of senescence in certain cell types. Therefore, novel therapeutic approaches are being actively explored. There is considerable hope vested in immunotherapy. Ongoing preclinical and clinical studies are evaluating RAS-targeted vaccines, adoptive T-cell therapy, PROTACs, and CRISPR/Cas technology-based strategies. However, further research is needed to exclude potential off-target effects and to find efficient delivery systems.

## Data Availability

No datasets were generated or analysed during the current study.

## Abbreviations

AML: Acute Myeloid Leukemia
ATC: Adoptive T-cell
AURKA: Aurora Kinase A
BRET: Bioluminescence Resonance Energy Transfer
CEA: Carcinoembryonic Antigen
COSMIC: The Catalog of Somatic Mutations in Cancer
CPG: Clinical Practice Guidelines
CRC: Colorectal Cancer
CRISPR: Clustered Regularly Interspaced Short Palindromic Repeats
DCR: Disease control rate
EGFR: Epidermal Growth Factor Receptor
ESMO: The European Society for Medical Oncology
FDA: The Food and Drug Administration
FDP: Farnesyl Diphosphate
FTase: Farnesyltransferase
FTI: Farnesyltransferase Inhibitor
GAP: GTPase Activating Protein
GDP: Guanosine 5′-diphosphate
HNSCC: Head and Neck Squamous Cell Carcinoma
ICGC: The International Cancer Genome Consortium
ICI: Immune Checkpoint Inhibitor
LODER: Local Drug EluteR
mCRC: Metastatic Colorectal Cancer
mDoR: Duration of Response
mPFS: Median Progression-Free Survival
MRD: Minimal Residual Disease
MSLN: Mesothelin
mTCR: Murine T-cell Receptor
NSAID: Nonsteroidal Anti-Inflammatory Drug
NSCLC: Non-Small Cell Lung Cancer
ORR: Objective Response Rate
PDAC: Pancreatic Ductal Adenocarcinoma
PKC: Protein Kinase C
POI: Protein of Interest
PPI: Protein‒Protein Interaction
PROTACs: PROteolysis TArgeting Chimeras
siRNA: Small Interfering RNA
TCGA: The Cancer Genome Atlas
TKI: Tyrosine Kinase Inhibitor
TRAEs: Treatment Emergent Adverse Events
